# National-scale changes in crop diversity through the Anthropocene

**DOI:** 10.1038/s41598-021-99728-5

**Published:** 2021-10-13

**Authors:** Rachel O. Mariani, Marc W. Cadotte, Marney E. Isaac, Denis Vile, Cyrille Violle, Adam R. Martin

**Affiliations:** 1 Department of Physical and Environmental Sciences, University of Toronto Scarborough, Toronto, Canada; 2 Department of Biological Sciences, University of Toronto Scarborough, Toronto, Canada; 3 Laboratoire d’Ecophysiologie des Plantes sous Stress Environnementaux (LEPSE, UMR759), Institut National de la Recherche Agronomique (INRA), Université de Montpellier, Montpellier, France; 4 CEFE, CNRS, EPHE, IRD, University Montpellier, University Paul Valéry, 34293 Montpellier, France

**Keywords:** Agroecology, Biodiversity, Sustainability

## Abstract

Expansion of crops beyond their centres of domestication is a defining feature of the Anthropocene Epoch. This process has fundamentally altered the diversity of croplands, with likely consequences for the ecological functioning and socio-economic stability of agriculture under environmental change. While changes in crop diversity through the Anthropocene have been quantified at large spatial scales, the patterns, drivers, and consequences of change in crop diversity and biogeography at national-scales remains less explored. We use production data on 339 crops, grown in over 150 countries from 1961 to 2017, to quantify changes in country-level crop richness and evenness. Virtually all countries globally have experienced significant increases in crop richness since 1961, with the early 1980s marking a clear onset of a ~ 9-year period of increase in crop richness in countries worldwide. While these changes have increased the similarity of diversity of croplands among countries, only half of countries experienced increases in crop evenness through time. Ubiquitous increases in crop richness within nearly all countries between 1980 and 2000 are a unique biogeographical feature of the Anthropocene. At the same time, we detected opposing changes in crop evenness, and only modest signatures of increased homogenization of croplands among countries. Therefore context-dependent and, at least, national-scale assessments are needed to understand and predict how changes in crop diversity influence agricultural resistance and resilience to environmental change.

## Introduction

The Anthropocene Epoch defines the current epoch of geologic time, where human activity is the dominant force shaping Earth’s abiotic and biotic environmental processes, systems, and cycles^1–3^. This novel geological epoch is defined in part by human-caused changes to Earth’s biogeography e.g.^4^, including the introduction of non-native or domesticated species e.g.^5^, and climate- and land-use change-induced shifts in species distributions e.g.^6^. However, arguably the most pronounced human-mediated changes in species biogeography defining the Anthropocene, albeit still largely understudied^7^, is the deliberate spread of crops outside of their centres of domestication into other parts of the world^8–10^. Large-scale anthropogenic influences on crop biogeography first emerged during the Columbian Exchange, which is a period in the early sixteenth century defined by massive exchanges of crop plants and animals between West Africa, Europe, and the Americas, during the time of New World colonization by Europeans^10^. During this time there was major movement of crops such as corn and beans from the Americas to Europe, while other crops such as wheat and barley were transported the opposite direction^9,10^.

While research defining the scientific basis of the Anthropocene have focused on the Columbian Exchange^2^, analyses of contemporary global food production trends have demonstrated that there have been more recent (i.e., post-1950) and widespread movement of new crops—i.e., those previously not present in large, industrial agricultural lands—into nearly all regions of the world^8^. Specifically since the early 1960s, the range of crops cultivated in agricultural lands across virtually all continents and regions, has changed in remarkably similar patterns: (1) in the 1960s continents experienced a period of little change in the number of new crops being cultivated in large (i.e., industrial) agricultural lands; this was followed by (2) a period of rapid increase in the number of crop groups being cultivated, beginning in the late 1970s and continuing through to the 1980s; and finally, (3) there exists a period of little change or levelling off in the number of crops being cultivated beginning in the 1990s and persisting through to present day^8^. These changes have drastically shaped not only the diversity of agricultural lands worldwide, but have greatly influenced global food supplies and security, diets, and agricultural economics^11–17^.

Coinciding with these human-mediated increases in the number of crops being cultivated in agricultural lands, this time period (particularly the 1980s) is also marked by an overall increase in the similarity of crop composition globally^8^. As an example of this homogenization of croplands at supra-national scales, wheat, maize, soy, and rice, now dominate over 50% of the world’s agricultural lands^18^. These changes have contributed to major shifts in the world’s agricultural landscapes and economies, including the homogenization of global food supplies and diets^14^, greater interdependency in agricultural trade between countries to maintain food security and potentially increases susceptibility of agricultural lands to pests, diseases, and climate stressors^19^.

Researchers have argued that the magnitude of these contemporary changes in crop biogeography, indicates that the 1980s are also a notable marker of the onset of the Anthropocene^8^. However, this aspect of a crop biogeography-based line of evidence supporting the Anthropocene hypothesis remains limited to research on changes in crop diversity at very large supranational regional, continental, or global spatial scales^8^, or a small number of national-scale analyses (e.g., within the United States^20^). This is despite reason to expect that the timing and rates of change in crop diversity is likely to differ widely among countries. For example previous work has shown that regional-scale agricultural policy initiatives including the Caribbean Basin Initiative and the North American Free Trade Agreement, were a primary catalyst for changes in crop diversity in the Caribbean and North American, respectively^8,21^. Yet such regional initiatives are not necessarily common in many agricultural economies. For instance, throughout regions of Africa, country-specific structural readjustment policies and colonial histories, but not necessarily region-scale policy initiatives, have played a key role in determining crop composition on agricultural lands^22^. Similarly, in certain countries Western philanthropic organizations have played a significant role in transferring crop technologies including disease resistant crops and pressuring shifts to cash crops, which have had unintended consequences for national agrobiodiversity^22,23^. This and a multitude of similar examples (e.g.,^21,24–26^) indicate that country-scale analyses are needed for a nuanced understanding of how crop diversity and composition has changed through the Anthropocene.

Supranational assessments also preclude nuanced analyses of the correlates or consequences of crop diversity change. Specifically, much of the structural adjustment policies that emphasized enhanced balance of trade and exports were imposed on the agricultural economies of developing nations throughout the 1980s e.g.^21,25^. Since these policies tended to focus on cultivation of new crops for international export markets (e.g., the introduction of cocoa in India and oil palm in Peru in the early and mid-1970s), one might expect that changes in agricultural diversity profiles (i.e., the timing, duration, and rate of change in crops being cultivated) vary systematically with socio-economic development indices. More specifically, one may hypothesize that a certain socio-economic group (e.g., developing nations with lower Human Development Index (HDI) values) to have broadly shown similar timing and rates of change in crop diversity, compared to countries with higher HDI.

Finally, one might expect that the number of crops cultivated within a given country also varies systemically with latitude, across a latitudinal gradient in crop diversity^15^. This pattern is likely to emerge as a function of multiple factors including (1) seasonality and limited growing conditions towards the poles^27^, and (2) the centres of crop domestication being disproportionately situated in tropical and sub-tropical regions^28,29^. Moreover, if a latitudinal gradient in the number of crops cultivated in agricultural lands does exist, these patterns may have been drastically altered by the incorporation of new crops into agricultural lands that began in the 1980s^8^.

National-scale analyses of crop cultivation are needed to test these questions surrounding crop diversity change, and its role in defining the Anthropocene. To address this, we used crop production data collected by the Food and Agricultural Organization (FAO)^18^ to execute national-scale analyses of changes in crop diversity. This analysis evaluated production data for 339 crop species groups, grown in 201 United Nations-recognized countries, over the past 56 years (from 1961 to 2017), resulting in a dataset of over ~ 2.36 million data points. Our analysis was designed to address the following: (1) How do patterns of change in the diversity of crops grown on agricultural lands, vary across countries in recent decades? (2) Have changes in country-scale crop diversity resulted in a detectable signal of “homogenization” across the world’s agricultural lands? (3) Are patterns of change in crop diversity across countries, systematically related to country-scale socio-economic indicators? (4) Does the number of crops cultivated across countries follow a latitudinal diversity gradient? And if so, (5) has the introduction of new crops into different countries altered this gradient?

## Results

### Changes in crop richness within countries through time

**Figure 1:**
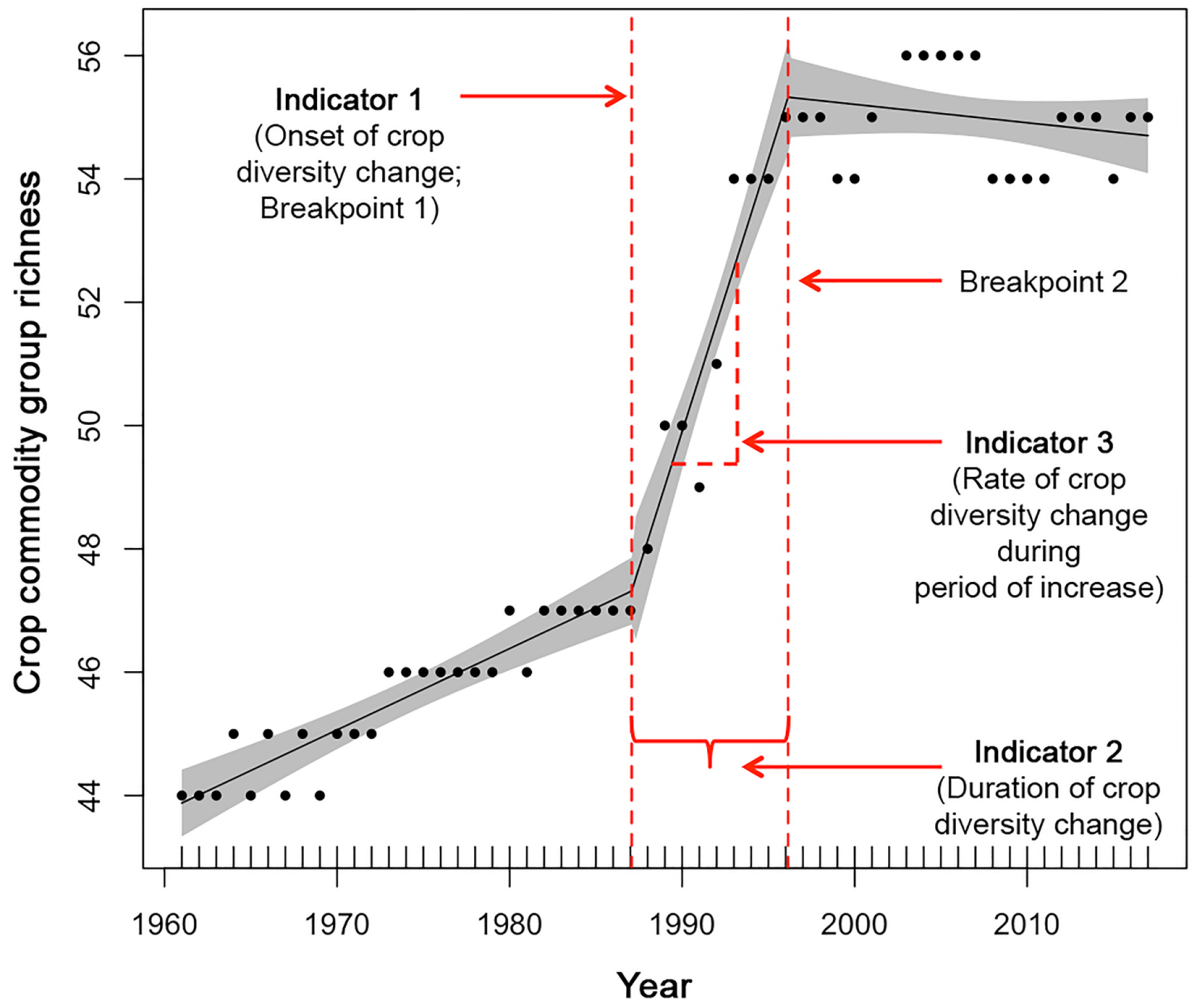
Schematic representation of three indicators of change in crop commodity group diversity, derived from piecewise models predicting crop commodity group richness as a function of year. Detailed explanations of Indicator 1–3 are presented in the Methods section. Data shown here as the example is from Canada, with black dots representing the number of commodities reported by the Food and Agricultural Organization, for a given year. Black trendline represents the piecewise model fit, gray bands represent the 95% confidence limits surrounding the model, and red lines represent model parameters and indicators derived from the model. *Note*: the figure presented here demonstrates changes in crop commodity group richness (*S*), though this framework was also employed for assessing change in crop group evenness (*J′*).

**Figure 2:**
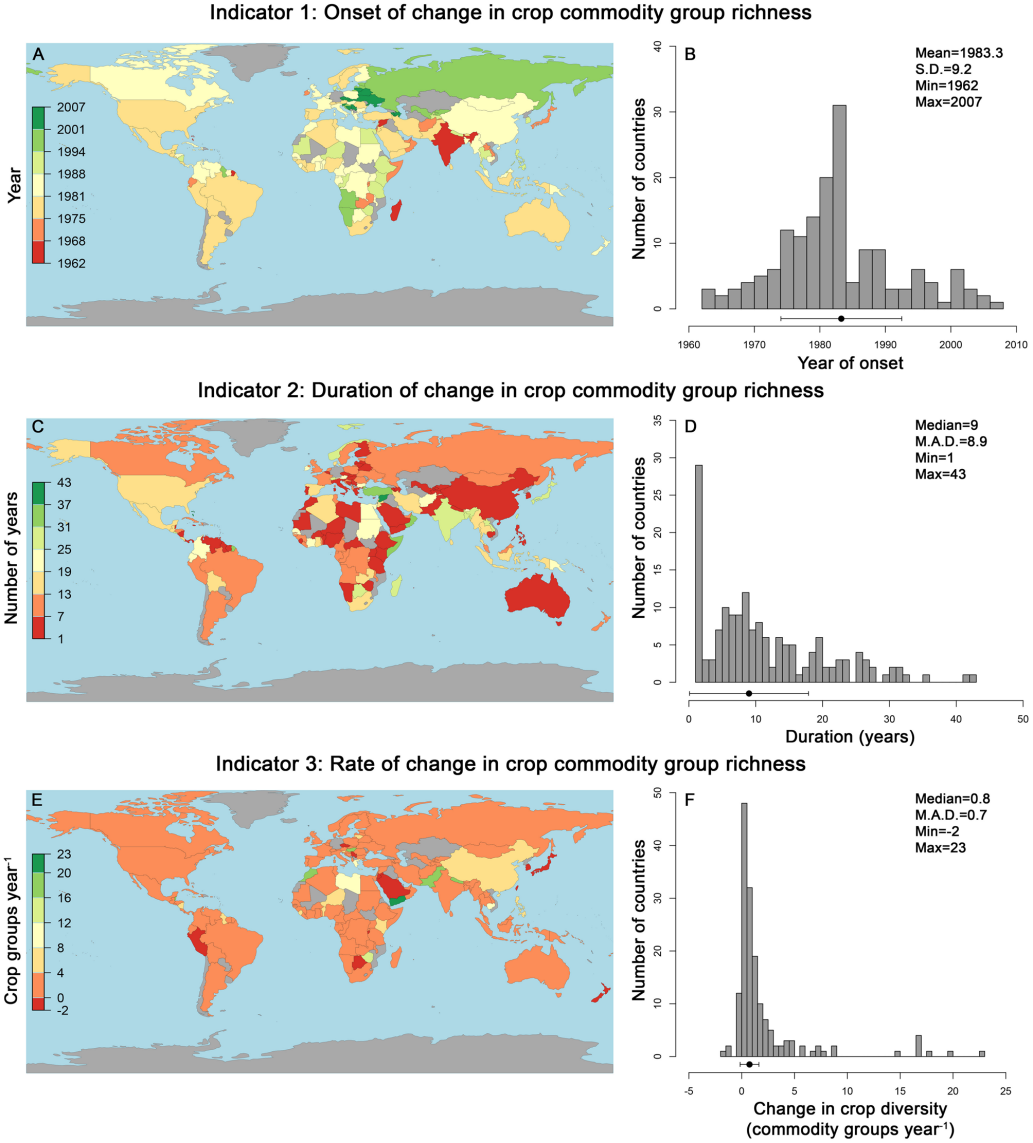
Maps and histograms of three indicators of crop commodity group richness (*S*) change across 165 countries. Values for all three indicators for each country were derived from piecewise linear models predicting *S* as a function of year (see Fig. 1 for example). Countries coloured gray in the maps were those where either data was not available or the piecewise models failed to converge (denoted in Table S1). Histograms and associated descriptive statistics for each indicator are also presented, with means (± s.d.) or medians (± m.a.d.) denoted visually by the points and error bars below the histograms. All piecewise model parameters for each country are presented in Table S1. Maps were generated using the mapCountryData function in the rworldmap R package^30^.

Our results are based on trends and three different “Indicator” values inferred from a piecewise modelling framework, which is described in detail in the Methods and presented conceptually in Fig. 1. Across all countries we detected significant changes, largely increases, in crop commodity group richness over time (*r*^2^ = 0.356–0.998 across 165 countries, *p* < 0.01 in all cases; Fig. 2, Table S1). Across 165 countries for which piecewise models converged, the average initial onset of crop group richness changes (Indicator 1) occurred in 1983 (± 9.2 years s.d.; Fig. 2A,B). The onset of changes in crop group richness occurred at the earliest in 1962 (in India), with the most recent onset of crop group richness change beginning in 2007 (in Serbia; Fig. 2A,B). Richness began to change at or after 1980 in 113 countries, of which 71% (or 80 countries) show changes in crop richness beginning from 1980 to 1989 (Fig. 2A; Table S1).

The period of crop richness change (Indicator 2) lasted approximately 9 years on average (± 8.9 years m.a.d.; Fig. 2C,D). Nineteen countries had a period of richness change lasting only one year, while nine countries experienced a more prolonged period of crop richness change lasting ≥ 30 years (Fig. 2C,D). National changes in richness began to saturate (i.e., ψ2 in Eq. (2), or breakpoint 2 in Fig. 1) on average in 1995 (± 8.3 years s.d.), with richness increases levelling off in 117 countries prior to the turn of the century at or before 1999 (Fig. 2C,D).

Over the period of change (i.e., between ψ1 and ψ2, Fig. 1), crop richness increased in 151 of 165 countries evaluated. Across all countries, richness increased (Indicator 3) on average by 0.8 species per year (± 0.9 m.a.d.; Fig. 2E,F). Only 13 countries reported decreases in richness per year, with these countries showing declines of 0.4 ± 0.6 crop groups per year on average (Fig. 2E,F, Table S1). In all of our datasets, Indicators 1–3 associated with changes in *S* were unrelated to the total area of a country (linear regression *r*^2^ = 0.0–0.004, *p* ≥ 0.888), or the area of a country under crop cultivation (linear regression *r*^2^ = 0.0–0.01, *p* ≥ 0.208).

**Figure 3:**
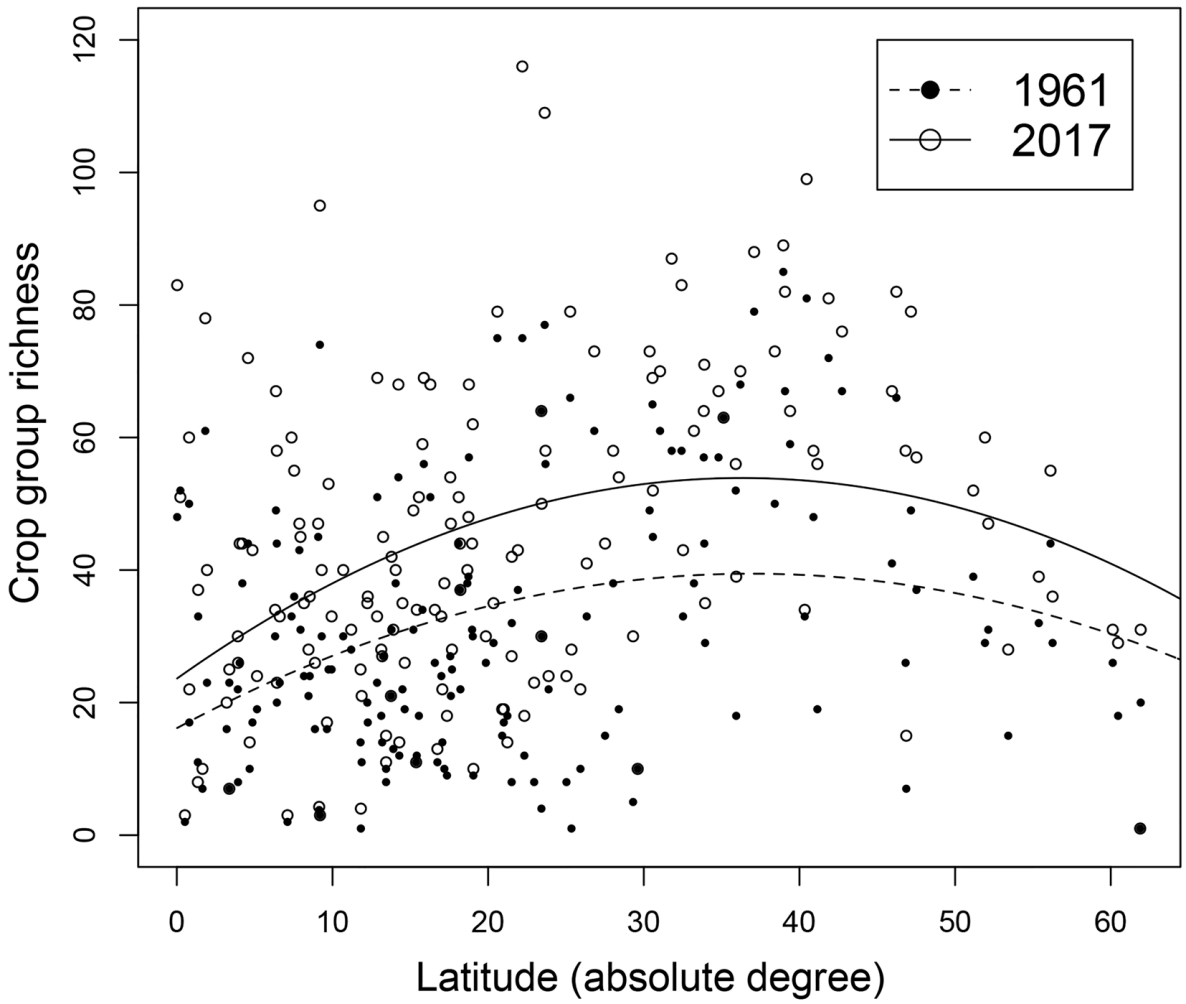
Latitudinal patterns in crop group richness across 164 countries in 1961 and 2017. Only countries with data from both years are included in this analysis. Trend lines represent results from linear models (including a 2nd-order polynomial term) that predict crop group richness as a function of latitude (and latitude^2^) in 1961 (dashed line, filled circles) and 2017 (solid line, open circles). Complete diagnostics for both models are presented in Table 1.

Across the 164 countries for which data was available in both 1961 and 2017, crop richness varied significantly as a function of latitude in similar patterns in both years (Table 1, Fig. 3). Latitude and a 2nd order polynomial term explained 10.5% and 12.9% of the variation in crop group richness in 1961 and 2017, respectively (model *p* ≤ 0.001 in both cases), with the richness-latitude relationship being similar in both years (Table 1). Specifically, regression models indicated that crop group richness increased from equatorial regions towards mid-latitude countries, with modeled peak crop group richness occurring at ~ 37° latitude in both 1961 and 2017; richness then declined at higher latitudes, denoted by statistically significant (*p* ≤ 0.002) negative 2nd order polynomial terms (Fig. 3). While these trends were similar between years, this analysis did reflect the increased crop group richness that occurred between 1961 and 2017, in 152 of 164 of the countries included in this analysis.

**Table 1:** Parameters and diagnostics for linear regression models (including a 2nd-order polynomial term) predicting crop group richness as a function of latitude in 1961 and 2017.

Year	Intercept	Latitude	Latitude^2^	Model *r*^2^ (*p* value)	Latitude with modelled peak richness
1961	16.1 (3.9)	1.3 (0.3)	− 0.02 (0.006)	0.105 (0.0001)	37.04°
2017	23.6 (4.5)	1.7 (0.4)	− 0.02 (0.006)	0.129 (< 0.0001)	36.39°

Only the 164 countries with data from both years were included in these analyses. Parameter estimate standard errors shown in parentheses. Also shown are the latitudes at which crop group richness peaked, according to these models, which are shown visually in Fig. 3.

### Changes in crop evenness and composition across countries through time

**Figure 4:**
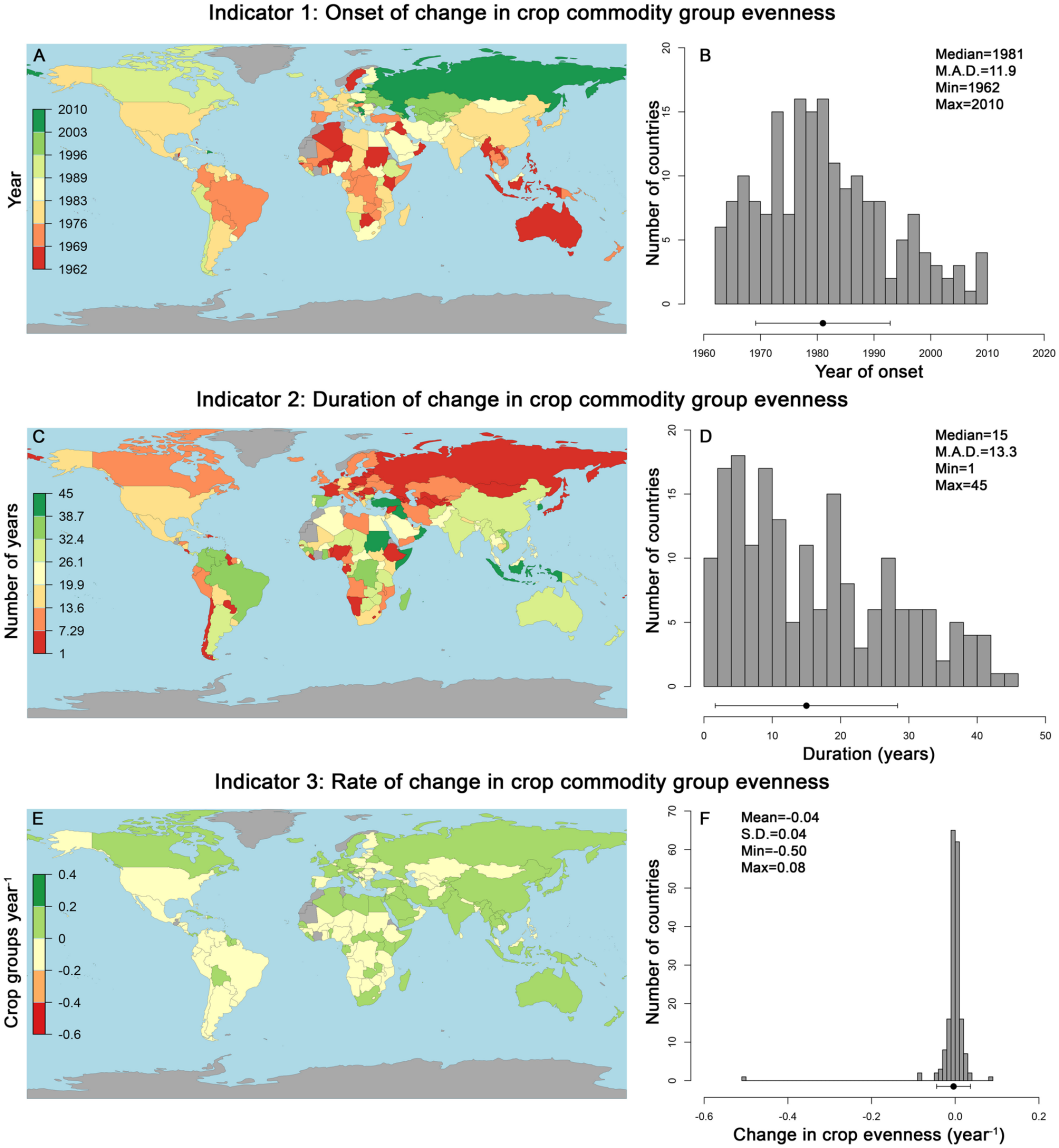
Maps and histograms of three indicators of crop commodity group evenness (Pielou’s evenness index (*J′*)) across 185 countries. Values for all three indicators for each country were derived from piecewise linear models predicting *J′* as a function of year, where harvested area (in ha) was used to approximate group abundance. Countries coloured gray in the maps were those where either data was not available or the piecewise models failed to converge (denoted in Table S2). Histograms and associated descriptive statistics for each indicator are also presented, with means (± s.d.) or medians (± m.a.d.) denoted visually by the points and error bars below the histograms. All piecewise model parameters for each country are presented in Table S2. Maps were generated using the mapCountryData function in the rworldmap R package^30^.

Piecewise models evaluating changes in crop group evenness (*J′*) through time converged for 185 countries; in these countries year explained on average 80.9% of the variation in *J′* (model *p* < 0.01 in all cases, *r*^2^ range = 0.198–0.992; Table S2). In these countries changes in *J′* began on average in 1981 (± 11.9 years m.a.d.), although compared to changes in group richness, periods of change in evenness were more prolonged lasting on average for 15 years (± 13.3 years m.a.d.) (Fig. 4A–D). Unlike analyses of crop richness, changes in evenness were less systematic, such that through the period of change evenness declined in 97 countries, increased in 88 countries, and average changes in *J′* centred on zero (mean = − 0.004 year^−1^ ± 0.04 s.d.; Fig. 4E,F). However, similar to patterns of change in richness, Indicators 1–3 associated with changes in evenness were also independent of total area of a country (linear regression *r*^2^ = 0.001–0.004, *p* ≥ 0.404), or the area of a country under crop cultivation (linear regression *r*^2^ = 0.0–0.013, *p* ≥ 0.12).

Multivariate analysis detected a significant influence of country, year, and a year-by-country interaction term on crop composition (Adonis *p* < 0.01 in all cases; Table 2). Of these variables, country differences were most pronounced with country identity explaining 89.5% of the variability in crop composition. Year explained ~ 1% of the variability in crop composition, while a country-by-year interaction term explained an additional 6.3% of the variation in crop composition (Table 2). Based on the non-metric multidimensional scaling (NMDS) analysis, there was trend of increasing similarity in crop composition among countries over time. This is illustrated by increasingly smaller 95% confidence bands surrounding the data points along NMDS axes 1 and 2 from 1961 through to 1983 (i.e., approximately the average year in which changes in crop group richness and evenness commence); multivariate space encapsulated by the 95% confidence band is then further reduced through 2017 (Figure S1).

**Table 2:** Results from an Adonis test evaluating changes in crop commodity group composition at national scales from 1961 to 2017.

Parameter	D.F	Sum of squares	Mean squares	Model *F*	*r*^2^	*p* value
Year	1	7.2	7.3	410.8	0.002	0.01
Country	175	3862.8	22.1	1250.8	0.895	0.01
Year*country	175	273.2	1.6	88.5	0.063	0.01
Residuals	9680	170.8	0.02	–	0.04	–
Total	10,031	4314.2	–	–	1	–

The distance matrix employed in this analysis was based on non-metric multidimensional scaling, whereby cultivation area was used as an estimate for crop group abundance. Results are presented visually in Figure S1.

### Socioeconomics correlates of changes in crop group diversity and evenness

Patterns of change in crop richness or evenness were not systematically related to country socio-economic status, with HDI values predicting only 1.3% of Indicators 1–3 for both *S* and *J′* on average (Table 3). The only exception to this is that the rate of change in *S* was significantly negatively related to HDI: countries with lower HDI scores expressed greater increases in *S* (i.e., Indicator 3 values) during their period of crop diversity change vs. countries with higher HDI (Table 3). This analysis did point to stronger explanatory power of spatial location in determining patterns of change in crop *S* and *J′*: both continent and region identity explained an average of 9.5% and 2.0% of the variation in Indicators 1–3, respectively (Table 3). However, ultimately country-by-country variation in crop group diversity change was largely idiosyncratic, with 87.0% of the variation in Indicators 1–3 for both *S* and *J′* being unaccounted for by socio-economic and spatial factors included in our models.

**Table 3:** Results and variance components predicting Indicators of crop group diversity (*S*) and evenness (*J′*) change, as a function of Human Development Index (HDI), continent identity, and region identity.

Variables		Fixed factors		Variance components (proportion explained)			
Metric	Indicator	Intercept	HDI	Fixed effects	Continent	Region	Unexplained
Crop group richness (S)	1	**1982.8 (5.0)**	2.0 (6.5)	0.001	0.101	0.074	0.825
	log-2	**1.8 (0.5)**	0.4 (0.6)	0.003	0.015	0.000	0.982
	log-3 + 10	**2.8 (0.1)**	− **0.5 (0.2)**	0.068	0.074	0.000	0.925
Crop group evenness (*J′*)	log-1	**7.6 (0.003)**	− 0.004 (0.004)	0.009	0.234	0.04	0.716
	log-2	**2.4 (0.5)**	0.2 (0.6)	0.001	0.138	0.007	0.855
	3	0.001 (0.01)	− 0.003 (0.01)	0.001	0.014	0.000	0.986

In these models an intercept and slope related to HDI were included as fixed effects, while region within continent were included as nested random effects. Statistically significant (*p* < 0.05) fixed effect model parameters are highlighted in bold, and data transformations (based on distribution fitting) precede the Indicator numbers (as per Figs. 2 and 4).

## Discussion

The vast majority of the world’s countries have experienced significant increases in the number of crop groups being cultivated over recent decades; changes that contribute to a detectable increase in the similarity of crops being grown among countries, that has occurred since the 1960s. Our findings align with those from previous studies^8^ that detected similar changes in crop taxonomic and phylogenetic diversity at supra-national regional, continental, and global scales: (1) a period of little change through the 1960s and 1970s, followed by (2) the onset of increases in crop commodity group richness commencing the 1980s and extending through the end of the 1990s, followed by (3) a levelling off of commodity group richness beyond the 2000s. While the concordance of these two studies should not be surprising, our findings contribute a new and more nuanced understanding of the remarkably similar patterns in crop group richness that have occurred across virtually all nations in recent decades; the consistency of which supports the idea that changes in crop diversity and biogeography occurring since the Columbian Exchange, are a ubiquitous feature of the Anthropocene.

However, these near-universal patterns in crop richness increases over recent decades (observed in all but 13 countries evaluated here) have had unequal and more variable effects on the evenness of crops being cultivated. The average duration of change in evenness (15 years) was nearly twice as long vs. the average duration of changes in richness (9 years). Previous work has noted that the majority of introduced crops into national agricultural production portfolios, largely owes to cultivation of crops beyond their country or region of origin^13^. So while introduction of novel crop groups has been rather succinct—corresponding to periods of rapid change in structural adjustment through the 1980s and 1990s^21,22^—expansion of these crops across more cultivated lands draws out over longer periods. This discrepancy likely reflects the lag between new crop introductions, compared to longer-term process associated with agricultural economic adjustments for these crops including expansion of export markets or crop-specific subsidies^15^, and to a much lesser extent, expansion of domestic consumption markets for new crops^11^.

The specific model parameters for country-specific trends may be sensitive to data quality^17^. However, here we observed a clear signal of both decreasing and increasing trends in crop evenness across 52% and 48% of the world’s countries, respectively. This approximately even proportion of increase and decrease in evenness detected among all countries here, would explain why analyses of global scale crop production trends reported no change in evenness between 1961 and 2013^11^. Yet similar to the growing number of analyses of production and consumption at multiple scales^8,11,14,15^, our multivariate analysis does indicate that over recent decades, countries have expressed a statistically significant increase in the similarity of crops in their agricultural lands. The implications of these shifts remain speculative, and based on our analysis, are clearly scale-dependent.

Specifically, increases in the similarity in crop composition across regions and continents could indicate growing susceptibility of agriculture to pest and pathogen outbreaks, and perhaps climate change effects like regional temperature increases or precipitation declines^8^. Alternatively, a wider geographic spread of crop groups could well represent a means of buffering production from localized disruptions including local climatic change or weather events, pest or pathogens, or civil unrest^11^. While both are plausible, generalizing either hypothesis to predict the impacts of homogenization across all agricultural lands globally is inconsistent with our country-specific analyses here. Since 1961, the evenness of crops in agricultural lands is both increasing and decreasing in approximately the same proportion of countries and agricultural area globally. Therefore, while previous studies including our own have speculated on how changes in crop diversity will influence global agricultural production and sustainability^8,11^, our results suggest the largest spatial scale at which potential impacts of stressors on crop production—though not food consumption or food security per se—can be robustly predicted is on a per-country basis.

Knowledge of the high context-dependency of agricultural adaptation, management, and crop selection likely indicates even smaller spatial scales (i.e., communities, households) are needed in order to fully predict susceptibility of production in the future. Indeed, an important caveat for our analysis is that even national scales likely do not comprehensively indicate how agricultural functional diversity has changed in the past 60 years. Our work here focuses on large-scale industrial agriculture, with the contributions of small-scale agriculture to overall crop diversity, particularly in terms of locally adapted varieties or landraces, likely underestimated^31^. Spatially explicit records of crop genetic diversity including locally adapted or cultivated crop phenotypes and their wild relatives are becoming more widely available, yet such a comprehensive assessment of crop genetic diversity remains prohibitive; indeed, while instructive, even analyses of phylogenetic crop diversity^8,32,33^ are limited to the crop species or sub-species levels. Clearly more work is needed to better integrate the methodological frameworks and concepts used here and in related studies^11,14^, with finer-scale datasets on crop genetic diversity.

### Determination of potential driving factors behind diversification trends

The factors underpinning national-scale increases in crop richness or evenness include a nuanced mix of environmental, socio-economic, and cultural factors. Here we hypothesized that patterns of change in crop group richness would correlate with development status (quantified as the Human Development Index). However, HDI was clearly a poor predictor of the rate, duration, and timing of changes in crop group richness and evenness. While national-scale agriculture portfolios did not systematically change as a function of HDI, spatial location (i.e., region and continent) did explain ~ 10–12% of the variation in patterns of change. From a strictly socio-economics perspective, this finding could point to regional-scale agricultural policy in driving similar changes in crop group richness, on a region-by-region basis^21,25^. Alternatively, it could also reflect regional-scale similarities in non-governmental organization (NGO) interventions in the agricultural sector^26^. Indeed, previous studies have indicated that governmental and NGO intervention has led to a ~ 17% increase in global crop diversity on average^26^. Additionally, and perhaps surprisingly, this same study^26^ found that while climate is an important driver of on-farm crop diversity change, its influence is secondary compared to market conditions.

### Agriculture and the reshuffling of species through the Anthropocene

We detected a statistically significant and hump-shaped latitudinal gradient in crop group richness, with low- to mid-latitude regions (centred on ~ 37° latitude), expressing the largest number of cultivated crop groups in large-scale agricultural lands. Consistent with previous studies, this non-linear latitudinal trend most likely emerges due to: (1) countries at these latitudes having a range of climatic conditions (i.e., Köppen climate zones) that supports cultivation of a large diversity of crop functional types and year-round production; and (2) countries at these latitudes encapsulating many of the world’s centres of crop domestication, and having received among the largest imports of crops during the Columbian Exchange^13,29,33^.

It is expected that the band of latitude supporting the highest number of crop groups could shift as a result of global environmental change drivers. Indeed, researchers have now long projected that crop diversity and richness within countries will change as climate change intensifies, with clusters of high crop diversity moving poleward through time^34,35^. However, similar to categorical comparisons of crop richness produced in tropical vs. temperature countries^15^, our analysis did not detect evidence of a disruption in the latitudinal trends of crop species richness in between 1961 and 2017. Instead, we find only a systematic increase in richness across all latitudes, indicating that the cultivation of new crop commodity groups in large-scale agricultural lands has not fundamentally altered the latitudes at which crop group richness is highest. Moreover, our analysis here (1) likely misses the role that high crop diversity of small holder (i.e. < 2-ha in size) farms^36^—which are disproportionately concentrated at lower latitudes^37^—play in driving latitudinal crop diversity gradients; and (2) does not address functional- or phylogenetic crop diversity, which may show different latitudinal patterns. Including these factors in additional analyses is a key step for further resolving our understanding of how crop biogeography is changing during the Anthropocene.

## Methods

### Data acquisition

Our analysis was based on open access crop production data from the United Nation’s Food and Agricultural Organization (FAO) spanning from 1961 to 2017^18^. We extracted data on area harvested (in ha) for 339 FAO-defined crop groups being grown in all UN-recognized countries. Since our research centred on understanding, quantifying, and mapping changes in crop diversity in current agricultural lands, countries that cease to exist (e.g., Yugoslavia) were not included in our analysis, resulting in data for 201 countries (Table S1). Prior to analyses, we adjusted certain crop group listings following our previous analyses of global changes in crop diversity^8^. Specifically, “Cottonlint” and “Cottonseed” were duplicated in our dataset and were therefore compiled as “Seedcotton”, while “Palmkernels” were renamed as “Oilpalmfruit.” Additionally, “Fruitpomenes”, “Fruitstonenes”, and “Grainmixed” were removed from analysis since these crop groupings are not associated with any specific crop species in the FAO database^18^. Finally, “Mushroomsandtruffles” were removed since it relates to non-plant species, and “Coir” was removed because it is a plant by-product.

### Changes in crop richness over time

All statistical analyses were performed using R version 3.3.3 statistical software (R Foundation for Statistical Computing, Vienna, Austria). The initial step in our analysis was to calculate both crop richness and evenness for each country, at each individual year, using the vegan R package^38^. Based on these datasets, we then used the analytical framework developed by^8^ to evaluate how crop species richness and evenness have changed in each individual country across its entire data range.

Specifically, in their analysis Martin et al.^8^ found that piecewise linear regression models provided the strongest descriptions of crop species richness change over time, across 21 of 22 FAO-defined regions globally. We therefore followed this approach by fitting a piecewise linear regression model for each country individually, that predicts changes in species richness over time. Piecewise model fitting was a two-step process, whereby for each country we first fit a linear regression model of the form:

1 $$S = a + \left( {b \times {\text{year}}} \right)$$

where *a* is the intercept and *b* represents the rate of change in crop group richness (*S*) through time. This linear model (Eq. 1) was then used as the basis of a piecewise linear regression model, which was fitted in order to estimate breakpoints in the relationship between *S* and year. Specifically, piecewise models were fit using the segmented function in the segmented R package^39^, and were of the form:

2 $$S = a + b\left( {{\text{year}}} \right) + \left( {\left( {c({\text{year}} -\uppsi _{1} } \right) \times I\left( {{\text{year}} >\uppsi _{1} } \right)} \right) + \left( {d\left( {{\text{year}} -\uppsi _{2} } \right) \times I\left( {{\text{year}} >\uppsi _{2} } \right)} \right)$$

where *a* is as in Eq. (1), and *b* represents the slope of the *S*-year relationship prior to the first breakpoint (ψ1). Here, *c* represents the difference in the slope of the *S*-year relationship between the first and second piecewise model segments; the *c* parameter therefore applies only when the first conditional indicator function (denoted by “*I*”) is true. Similarly, *d* represents the difference in slopes for the *S*-year relationship between the first, second, and third segments, which only applies when the second conditional indicator function is true. In sum, the slope of the relationship between *S* and year is equal to *b* prior to the ψ1, is equal to *b* + *c* between ψ1and ψ2, and is equal to *b* + *c* + *d* after ψ2. Piecewise models were fit with initial starting parameters of 1975 and 2000 for ψ1 and ψ2, respectively. The ψ1 and ψ2 parameters were tuned manually for 29 countries with a shortened data range, following visual inspection of data (see Tables S1 and S2).

Based on this piecewise regression model procedure, we then used parameters from Eq. (2) to determine three key indicator points of crop diversity change through time for each country (displayed visually in Fig. 1). Indicator 1 reflects the onset of diversification in each country, and was calculated as Breakpoint 1 (ψ1) in Eq. (2); this indicator therefore corresponds to the year in which notable changes in species richness began. Indicator 2 reflects the duration of the crop diversification period in each country, and was calculated as the difference between breakpoints 2 and 1 (i.e., ψ2-ψ1 from Eq. 2); this indicator therefore represents the duration of the period when crop prominent changes in crop diversity occurred. Finally, Indicator 3 reflects the rate at which crop diversity changed throughout the diversification period in each country; this indicator was calculated as the rate of crop diversity change (between ψ1 and ψ2), which in our models corresponded to the sum of the slopes (1) prior to the first breakpoint, and (2) between the first and second breakpoints (i.e., corresponding to *b* + *c* in Eq. 2). For each indicator we then calculated summary statistics as either mean ± standard deviations or median ± median absolute deviations (m.a.d.), where data was normally or log-normally distributed, respectively. Country values for each indicator were mapped using the mapCountryData function in the rworldmap R package^40^.

### Changes in crop evenness over time

Evaluations of temporal changes in crop evenness at national scales followed this same analytical approach as above. First, for each country-by-year combination we calculated Pielou's evenness index (*J′*)—which ranges from 0 to 1, with values closer to 0 indicating less evenness or greater abundance of a few dominant crop groups, and values closer to 1 representing more equitable abundances of crop groups—as:

3 $$J^{\prime} = \frac{H^\prime }{{\ln \left( S \right)}}$$

where *S* is again crop richness, and *H′* is the Shannon–Weiner diversity index calculated as:

4 $$H^\prime = - \mathop \sum \limits_{i = 1}^{S} p_{i} ln p_{i}$$

where *p*_*i*_ represents the relative proportion of the *i*th crop group for a given country-by-year combination. In these evenness calculations, all values of *p*_*i*_ were estimated as the relative proportion of agricultural area (measured in ha) occupied by a given crop commodity group, within a country at a given year; this analytical approach was employed by Martin et al.^8^ when assessing crop group composition at supra-national scales. We then evaluated how *J′* values changed in each country through time by replicating our stepwise modelling analyses above, substituting *J′* for *S* in Eqs. (1) and (2), and extracting the same model indicators (Fig. 1). Finally, we calculated summary statistics and mapped each of these indicators, as described above.

### Changes in crop composition across countries and over time

We used multivariate analyses to evaluate how temporal changes in *S* and *J′* influenced crop composition across countries and over time. To do so, we created a community composition matrix whereby national-level crop assemblages were estimated for each of the country-by-year combinations. In this matrix, area harvested was taken as an approximation of the abundance of each crop group within each country-by-year combination (again following Martin et al.^8^). Since these abundances (or area harvested) across country-by-year combinations varied over orders of magnitude, we used non-metric multidimensional scaling (NMDS) to analyze and visualize spatial (country) and temporal (year) differences in crop diversity. Specifically, we used the vegan R package^38^ to calculate all 58,899,231 Bray–Curtis dissimilarities among all 10,854 data points (i.e., crop group composition in every country-by-year data point), as:

5 $$BC_{jk} = \frac{{\sum i \left| {x_{ij} - x_{ik} } \right|}}{{\sum i \left( {x_{ij} + x_{ik} } \right)}}$$

where *BC*_*jk*_ represents the dissimilarity between the *j*th and *k*th community, *x*_*ij*_ represents the abundance (i.e., area harvested) of crop group *i* in sample *j*, and *x*_*ik*_ represents the abundance of crop group *i* in sample *k*. We then used a multivariate analysis of variance (i.e., an Adonis test), to test for significant differences in Bray–Curtis distances as a function of country, year, and a country-by-year interaction. Significance was assessed using a permutation test, with 99 permutations used.

### Latitudinal gradients in crop richness

To test our hypotheses surrounding the presence of, and temporal changes in, latitudinal gradients in crop group diversity, we focused on 164 countries for which crop group diversity was available in both 1961 and 2017. For each of these two datasets, we fit a separate linear regression model that predicts crop group richness as a function of latitude (expressed as an absolute value) and a 2nd-order polynomial term for the ‘latitude^2^’ variable. From both of these models, we extracted and compared latitude value at which crop group richness was estimated/ modelled to peak.

### Predictors of change in crop diversity and composition

We tested if Human Development Index (HDI) was correlated with patterns of change in crop diversity and composition. Briefly, the HDI is a composite index of four metrics related to socio-economic status, including life expectancy at birth, expected years of schooling for children at a school-centring age, mean years of schooling for adults ≥ 25 years of age, and log-transformed gross national income per capita. These values are then aggregated on a per country basis, into an HDI index that ranges from 0–1 with higher scores denoting higher performance in these indicators. We employed 2017 HDI values in our analysis here, in order to include the most countries possible in each analysis (since earlier HDI scores are less readily available)^41^.

We then used linear mixed effects models to test if patterns of change in crop diversity and evenness varied systematically with HDI values. This entailed fitting six linear mixed models, where each of our six indicators (i.e., Indicators 1–3 for both *S* and *J′*) were predicted as a function of HDI; these models also accounted for potential spatial autocorrelation in Indicator values by including the FAO-defined continent identity and FAO-defined region identity of each country, as a nested random variable. Models were fit using the lme function in the nlme R package^41^. We then estimated the proportion of variation in each indicator that is explained by HDI, continent identity, and region identity, using the varcomp function in the ape R package^42^—which partitioned explained variation across continents and regions—as well as the sem.model.fits function in the piecewiseSEM R package^43^—which partitioned explained variation across the fixed (i.e., model intercept and HDI) vs. random (i.e., continent and region) effects. Due to differences in HDI data availability and in the number of piecewise models that converged, *n* = 152 countries for all models of *S* indicators and *n* = 139 countries for all models of *J′* indicators. Log-transformed values of Indicators were used in these analyses where they better approximated a log-normal distribution, as determined using the fitdistrplus function in the fitdistrplus R package^44^.

## Supplementary Information

Supplementary Information.

## References

[1] Crutzen PJ (2006). Earth System Science in the Anthropocene.

[2] Lewis SL, Maslin MA (2015). Defining the Anthropocene. Nature.

[3] Malhi Y (2017). The concept of the Anthropocene. Annu. Rev. Environ. Resour..

[4] Young KR (2014). Biogeography of the Anthropocene: Novel species assemblages. Prog. Phys. Geogr..

[5] Capinha C, Essl F, Seebens H, Moser D, Pereira HM (2015). The dispersal of alien species redefines biogeography in the Anthropocene. Science.

[6] Parmesan C, Yohe G (2003). A globally coherent fingerprint of climate change impacts across natural systems. Nature.

[7] Zimmerer KS (2019). The biodiversity of food and agriculture (Agrobiodiversity) in the anthropocene: Research advances and conceptual framework. Anthropocene.

[8] Martin AR (2019). Regional and global shifts in crop diversity through the Anthropocene. PLoS ONE.

[9] Crosby AW (2003). The Columbian Exchange: Biological and Cultural Consequences of 1492.

[10] Mann, C. C. *1493: How the ecological collision of Europe and the Americas gave rise to the modern world* (2011).

[11] Aguiar S, Texeira M, Garibaldi LA, Jobbágy EG (2020). Global changes in crop diversity: Trade rather than production enriches supply. Glob. Food Secur..

[12] Campi M, Dueñas M, Fagiolo G (2021). Specialization in food production affects global food security and food systems sustainability. World Dev..

[13] Khoury CK (2016). Origins of food crops connect countries worldwide. Proc. R. Soc. B Biol. Sci..

[14] Khoury CK (2014). Increasing homogeneity in global food supplies and the implications for food security. Proc. Natl. Acad. Sci. U.S.A..

[15] Nelson EJ (2016). Commercial plant production and consumption still follow the latitudinal gradient in species diversity despite economic globalization. PLoS ONE.

[16] Renard D, Tilman D (2019). National food production stabilized by crop diversity. Nature.

[17] Mahaut L, Violle C, Renard D (2021). Complementary mechanisms stabilize national food production. Sci. Rep..

[18] Nations, F. A. O. U. http://www.fao.org/faostat/en/ (2020).

[19] Hessenauer P (2021). Evolution and adaptation of forest and crop pathogens in the Anthropocene. Phytopathology.

[20] Aguilar J (2015). Crop species diversity changes in the United States: 1978–2012. PLoS ONE.

[21] Isakson SR (2014). Maize diversity and the political economy of agrarian restructuring in Guatemala. J. Agrar. Chang..

[22] Bonneuil C (2000). Development as experiment: Science and state building in late colonial and postcolonial Africa, 1930–1970. Osiris.

[23] Holt-Gimenez, E., Altieri, M. A. & Rosset, P. Food First Policy Brief No. 12: Ten Reasons Why the Rockefeller and the Bill and Melinda Gates Foundations' Alliance for Another Green Revolution Will Not Solve the Problems of Poverty and Hunger in Sub-Saharan Africa. (Institute of Food and Development Policy, Oakland, USA, 2006).

[24] Arnold D (2005). Europe, technology, and colonialism in the 20th century. Hist. Technol..

[25] Meertens B (2000). Agricultural performance in Tanzania under structural adjustment programs: Is it really so positive?. Agric. Hum. Values.

[26] Chen M (2018). Diversification and intensification of agricultural adaptation from global to local scales. PLoS ONE.

[27] Pontarp M (2019). The latitudinal diversity gradient: Novel understanding through mechanistic eco-evolutionary models. Trends Ecol. Evol..

[28] Harlan JR (1971). Agricultural origins: Centers and noncenters. Science.

[29] Meyer RS, DuVal AE, Jensen HR (2012). Patterns and processes in crop domestication: An historical review and quantitative analysis of 203 global food crops. New Phytol..

[30] South A (2011). rworldmap: A new R package for mapping global data. R J..

[31] Ricciardi, V., Mehrabi, Z., Wittman, H., James, D. & Ramankutty, N. Higher yields and more biodiversity on smaller farms. *Nat. Sustain.* 1–7, (2021).

[32] Milla R (2020). Crop origins and phylo food: A database and a phylogenetic tree to stimulate comparative analyses on the origins of food crops. Glob. Ecol. Biogeogr..

[33] Milla R (2018). Phylogenetic patterns and phenotypic profiles of the species of plants and mammals farmed for food. Nat. Ecol. Evol..

[34] Leemans, R. & Solomon, A. M. Modeling the potential change in yield and distribution of the earth's crops under a warmed climate. *Clim. Res.* 79–96 (1993).

[35] Zabel F, Putzenlechner B, Mauser W (2014). Global agricultural land resources–a high resolution suitability evaluation and its perspectives until 2100 under climate change conditions. PLoS ONE.

[36] Jarvis DI (2008). A global perspective of the richness and evenness of traditional crop-variety diversity maintained by farming communities. Proc. Natl. Acad. Sci. U.S.A..

[37] Lowder SK, Skoet J, Raney T (2016). The number, size, and distribution of farms, smallholder farms, and family farms worldwide. World Dev..

[38] Oksanen, J. *et al.* vegan: Community Ecology Package. R package version 2.4–5. (2017).

[39] Vito M, Muggeo R (2008). Segmented: An R package to fit regression models with broken-line relationships. cran.r-project.org/doc/Rnews/. R News.

[40] South A (2011). rworldmap: A new R PACKAGE FOR MAPPING GLOBAL DATa. The R Journal.

[41] nlme: Linear and Nonlinear Mixed Effects Models. R package version 3.1–131 (https://CRAN.R-project.org/package=nlme, 2017).

[42] Paradis E, Claude J, Strimmer K (2004). APE: Analyses of phylogenetics and evolution in R language. Bioinformatics.

[43] Lefcheck JS (2016). piecewiseSEM: Piecewise structural equation modelling in R for ecology, evolution, and systematics. Methods Ecol. Evol..

[44] Delignette-Muller ML, Dutang C (2015). fitdistrplus: An R package for fitting distributions. J. Stat. Softw..

