# Supplementary material for: National-scale changes in crop diversity through the Anthropocene
Source: Sci Rep. 2021 Oct 13;11:20361. doi: 10.1038/s41598-021-99728-5 (PMC8514462; doi:10.1038/s41598-021-99728-5)
Supplement: Supplementary file 1 — Supplementary Information. [file 41598_2021_99728_MOESM1_ESM.docx]

**Supplementary Information for:**

National-scale changes in crop diversity through the Anthropocene

**Authors and Affiliations:** Rachel O. Mariani, Marc W. Cadotte^2^, Marney E. Isaac^1^, Adam R. Martin^1,*^

^1^ Department of Physical and Environmental Sciences and The Centre for Critical Development Studies, University of­­­ Toronto Scarborough, Canada.

^2^ Department of Biologic­­­al Sciences, University of Toronto Scarborough, Canada. ­

* Corresponding author contact: adam.martin@utoronto.ca

**Table S1.** Final piecewise modeling results for the 201 countries included in our analysis of temporal changes in crop richness. Presented here are six parameters fitted for each country-specific piecewise model (see Equation 2), piecewise model *r*^2^ values, along with three indicators were calculated for each country (based on Equation 2, visualized in Figure 1). Piecewise models converged and were fitted to 165 countries. The 36 countries were model parameters, diagnostics, and indicators are denoted as “-”, represent the countries where piecewise models did not converge. In these instances, models for 10 countries did not converge because these countries reported no change in crop commodity group richness through time (denoted by “*” beside these country names). In the remaining 26 countries (denoted by “**” beside these country names) crop commodity group richness increased through time, between the first and last year of data. However, in these 26 countries, increases in crop commodity group richness were primarily reflected by a single step increase; these changes could not be fitted through our piecewise modelling procedure.

|  | **Piecewise model parameters** | | | | | |  | **Indicators** | | |
| --- | --- | --- | --- | --- | --- | --- | --- | --- | --- | --- |
| **Country** | ***a*** | ***b*** | ***c*** | ***d*** | **ψ1** | **ψ2** | **Model *r*^2^** | **Indicator 1** | **Indicator 2** | **Indicator 3** |
| Afghanistan | 29.00 | 0.00 | 0.25 | -0.25 | 1970 | 1994 | 0.964 | 1970 | 24 | 0.25 |
| Albania | -44.32 | 0.03 | 2.95 | -2.45 | 1983 | 1992 | 0.987 | 1983 | 9 | 2.98 |
| Algeria | -1450.63 | 0.76 | -0.32 | -0.41 | 1980 | 1996 | 0.986 | 1980 | 16 | 0.44 |
| American Samoa** | - | - | - | - | - | - | - | - | - | - |
| Angola | 28.00 | 0.00 | 0.26 | -0.26 | 1996 | 2008 | 0.965 | 1996 | 12 | 0.26 |
| Antigua and Barb. | -1543.00 | 0.79 | -0.63 | -0.17 | 1966 | 1994 | 0.874 | 1966 | 28 | 0.17 |
| Argentina | -105.89 | 0.08 | 2.53 | -2.61 | 1978 | 1987 | 0.984 | 1978 | 9 | 2.61 |
| Armenia | -386.46 | 0.21 | 3.79 | -3.94 | 2003 | 2004 | 0.992 | 2003 | 1 | 4.00 |
| Australia | 176.02 | -0.06 | 1.81 | -1.70 | 1979 | 1986 | 0.970 | 1979 | 7 | 1.75 |
| Austria | 780.12 | -0.38 | 1.49 | -1.00 | 1980 | 2004 | 0.968 | 1980 | 24 | 1.12 |
| Azerbaijan | 122.92 | -0.03 | 1.12 | -0.98 | 2003 | 2008 | 0.948 | 2003 | 5 | 1.09 |
| Bahamas | 5.52 | 0.00 | 0.11 | 1.22 | 1963 | 2006 | 0.900 | 1963 | 43 | 0.11 |
| Bahrain | 10.00 | 0.00 | 4.50 | -4.47 | 1973 | 1975 | 0.991 | 1973 | 2 | 4.50 |
| Bangladesh | 56.00 | 0.00 | 0.08 | -0.08 | 1984 | 2010 | 0.938 | 1984 | 26 | 0.08 |
| Barbados | 14.00 | 0.00 | 0.98 | -0.95 | 1980 | 1992 | 0.991 | 1980 | 12 | 0.98 |
| Belarus | -404.73 | 0.22 | 2.87 | -3.17 | 2002 | 2004 | 0.963 | 2002 | 2 | 3.08 |
| Belgium | -199.94 | 0.12 | 0.80 | -0.92 | 1983 | 1989 | 0.972 | 1983 | 6 | 0.92 |
| Belize | -119.82 | 0.07 | 17.93 | -17.70 | 1988 | 1989 | 0.994 | 1988 | 1 | 18.00 |
| Benin | 8.61 | 0.01 | 6.99 | -6.92 | 1984 | 1985 | 0.992 | 1984 | 1 | 7.00 |
| Bermuda* | - | - | - | - | - | - | - | - | - | - |
| Bhutan | -174.17 | 0.10 | 0.40 | 0.42 | 1975 | 1998 | 0.963 | 1975 | 23 | 0.50 |
| Bolivia | -362.35 | 0.21 | 0.62 | -0.79 | 1975 | 1991 | 0.989 | 1975 | 16 | 0.83 |
| Bosnia and Herz. | -52.00 | 0.05 | 1.24 | -1.22 | 2001 | 2008 | 0.991 | 2001 | 7 | 1.29 |
| Botswana | -198.42 | 0.11 | -0.13 | 1.53 | 1982 | 2013 | 0.856 | 1982 | 31 | -0.03 |
| Brazil | -219.14 | 0.14 | 0.98 | -1.06 | 1980 | 1990 | 0.989 | 1980 | 10 | 1.12 |
| British Virgin Is. | -289.59 | 0.15 | -0.10 | -0.05 | 1967 | 1993 | 0.898 | 1967 | 26 | 0.05 |
| Brunei | -392.18 | 0.21 | 0.76 | -1.11 | 1990 | 2000 | 0.821 | 1990 | 10 | 0.97 |
| Bulgaria | 725.35 | -0.33 | 1.04 | -0.74 | 1985 | 2007 | 0.938 | 1985 | 22 | 0.71 |
| Burkina Faso | -310.83 | 0.17 | 3.83 | -3.96 | 1983 | 1985 | 0.993 | 1983 | 2 | 4.00 |
| Burundi | -405.31 | 0.22 | -0.33 | 0.16 | 1971 | 1980 | 0.815 | 1971 | 9 | -0.12 |
| Cambodia | 24.37 | 0.00 | 8.25 | -8.20 | 1984 | 1985 | 0.992 | 1984 | 1 | 8.25 |
| Cameroon | -332.19 | 0.19 | 2.03 | -2.11 | 1982 | 1992 | 0.990 | 1982 | 10 | 2.22 |
| Canada | -214.72 | 0.13 | 0.75 | -0.91 | 1987 | 1996 | 0.977 | 1987 | 9 | 0.88 |
| Cape Verde** | - | - | - | - | - | - | - | - | - | - |
| Cayman Is. | -2102.02 | 1.07 | -0.98 | -0.09 | 1968 | 1982 | 0.902 | 1968 | 14 | 0.09 |
| Central African Rep. | 2.52 | 0.01 | 2.99 | -2.98 | 1983 | 1986 | 0.990 | 1983 | 3 | 3.00 |
| Chad** | - | - | - | - | - | - | - | - | - | - |
| Chile** | - | - | - | - | - | - | - | - | - | - |
| China | -47.37 | 0.06 | 7.24 | -6.92 | 1983 | 1987 | 0.986 | 1983 | 4 | 7.30 |
| Colombia | -75.48 | 0.06 | 1.22 | -1.18 | 1984 | 2004 | 0.961 | 1984 | 20 | 1.28 |
| Comoros | 139.09 | -0.07 | 1.40 | -1.33 | 1983 | 1992 | 0.981 | 1983 | 9 | 1.33 |
| Republic of Congo | 26.00 | 0.00 | 1.71 | -1.68 | 1988 | 1998 | 0.988 | 1988 | 10 | 1.71 |
| Cook Is. | -50.17 | 0.04 | -0.14 | -0.11 | 1982 | 1998 | 0.957 | 1982 | 16 | -0.11 |
| Costa Rica | -6.05 | 0.02 | 1.46 | -1.48 | 1979 | 1998 | 0.996 | 1979 | 19 | 1.48 |
| Ivory Coast | -204.00 | 0.12 | 0.77 | -0.89 | 1981 | 2001 | 0.991 | 1981 | 20 | 0.89 |
| Croatia | -204.00 | -0.25 | 0.45 | -0.11 | 1998 | 2010 | 0.722 | 1998 | 12 | 0.19 |
| Cuba | 19.11 | 0.01 | 1.03 | -0.98 | 1982 | 1991 | 0.980 | 1982 | 9 | 1.03 |
| Cyprus | 283.76 | -0.11 | 1.11 | -1.01 | 1984 | 1986 | 0.356 | 1984 | 2 | 1.00 |
| Czech Rep. | -41.77 | 0.05 | -1.05 | 1.00 | 2006 | 2007 | 0.627 | 2006 | 1 | -1.00 |
| Democratic Republic of Congo | 309.36 | -0.13 | 0.26 | -0.13 | 1982 | 1993 | 0.862 | 1982 | 11 | 0.13 |
| Denmark | 105.13 | -0.04 | 0.71 | -1.57 | 1994 | 2013 | 0.885 | 1994 | 19 | 0.67 |
| Djibouti | 1.00 | 0.00 | 0.24 | -0.24 | 1978 | 1991 | 0.990 | 1978 | 13 | 0.24 |
| Dominica | -31.36 | 0.02 | 1.75 | -1.68 | 1976 | 1987 | 0.985 | 1976 | 11 | 1.77 |
| Dominican Rep.** | - | - | - | - | - | - | - | - | - | - |
| Ecuador | 61.00 | 0.00 | 0.82 | -0.76 | 1973 | 1993 | 0.989 | 1973 | 20 | 0.82 |
| Egypt | -770.99 | 0.42 | -0.35 | -0.29 | 1991 | 2011 | 0.990 | 1991 | 20 | 0.08 |
| El Salvador | -124.30 | 0.08 | 0.25 | -0.33 | 1979 | 2009 | 0.968 | 1979 | 30 | 0.33 |
| Eq. Guinea | 7.00 | 0.00 | 0.33 | -0.33 | 1984 | 1993 | 0.987 | 1984 | 9 | 0.33 |
| Eritrea* | - | - | - | - | - | - | - | - | - | - |
| Estonia | 204.79 | -0.09 | 0.38 | -0.43 | 2000 | 2008 | 0.841 | 2000 | 8 | 0.30 |
| Ethiopia | -7914.00 | 4.00 | -2.71 | -1.29 | 1994 | 2000 | 0.987 | 1994 | 6 | 1.29 |
| Faroe Is.* | - | - | - | - | - | - | - | - | - | - |
| Fiji | 7.23 | 0.01 | 0.64 | -0.71 | 1984 | 1996 | 0.973 | 1984 | 12 | 0.65 |
| Finland | -23.27 | 0.02 | 1.30 | -1.25 | 1987 | 1994 | 0.992 | 1987 | 7 | 1.32 |
| France | -269.28 | 0.17 | 1.21 | -1.33 | 1984 | 1992 | 0.990 | 1984 | 8 | 1.38 |
| French Guiana | 8.00 | 0.00 | 0.49 | -0.45 | 1964 | 2000 | 0.951 | 1964 | 36 | 0.49 |
| Fr. Polynesia | 25.00 | 0.00 | 0.13 | -0.13 | 1970 | 1993 | 0.932 | 1970 | 23 | 0.13 |
| Gabon | 17.00 | 0.00 | 0.43 | -0.43 | 1983 | 1994 | 0.990 | 1983 | 11 | 0.43 |
| Gambia** | -112.01 | 0.06 | 0.24 | -0.25 | 1983 | 1991 | 0.964 | 1983 | 8 | 0.31 |
| Georgia** | - | - | - | - | - | - | - | - | - | - |
| Germany | - | - | - | - | - | - | - | - | - | - |
| Ghana | -73.66 | 0.05 | 0.69 | -0.75 | 1984 | 2001 | 0.976 | 1984 | 17 | 0.75 |
| Greece | 74.79 | 0.00 | 9.00 | -8.82 | 1984 | 1985 | 0.990 | 1984 | 1 | 9.00 |
| Grenada | -957.60 | 0.49 | 0.37 | -0.67 | 1988 | 1995 | 0.863 | 1988 | 7 | 0.87 |
| Guadeloupe | -299.84 | 0.16 | 0.47 | -0.69 | 1984 | 1995 | 0.821 | 1984 | 11 | 0.63 |
| Guam | -2.11 | 0.01 | 0.73 | -0.75 | 1980 | 1985 | 0.965 | 1980 | 5 | 0.73 |
| Guatemala | -124.90 | 0.08 | 1.30 | -1.38 | 1976 | 1994 | 0.990 | 1976 | 18 | 1.38 |
| Guinea | -85.85 | 0.06 | 0.44 | -0.39 | 1981 | 1990 | 0.986 | 1981 | 9 | 0.50 |
| Guinea Bissau | 14.02 | 0.00 | 0.76 | -0.67 | 1975 | 1986 | 0.991 | 1975 | 11 | 0.76 |
| Guyana | -372.53 | 0.20 | 4.00 | -4.22 | 1995 | 2000 | 0.986 | 1995 | 5 | 4.20 |
| Haiti | 31.00 | 0.00 | 1.62 | -1.62 | 1982 | 1990 | 0.980 | 1982 | 8 | 1.62 |
| Honduras | 31.00 | 0.00 | 1.97 | -1.97 | 1983 | 1993 | 0.991 | 1983 | 10 | 1.97 |
| Hungary | -149.98 | 0.10 | 16.90 | -16.67 | 1984 | 1985 | 0.981 | 1984 | 1 | 17.00 |
| Iceland* | - | - | - | - | - | - | - | - | - | - |
| India | -1253.09 | 0.68 | -0.55 | -0.13 | 1962 | 1988 | 0.973 | 1962 | 26 | 0.13 |
| Indonesia | -51.17 | 0.05 | 0.64 | -0.69 | 1977 | 1991 | 0.989 | 1977 | 14 | 0.69 |
| Iran | 65.12 | 0.00 | 1.37 | -1.32 | 1980 | 1996 | 0.968 | 1980 | 16 | 1.37 |
| Iraq** | - | - | - | - | - | - | - | - | - | - |
| Ireland | 15.00 | 0.00 | 0.62 | -0.62 | 1972 | 1993 | 0.991 | 1972 | 21 | 0.62 |
| Israel | -221.98 | 0.14 | 0.17 | -0.24 | 1971 | 1991 | 0.929 | 1971 | 20 | 0.31 |
| Italy | 138.62 | -0.03 | 3.11 | -3.04 | 1983 | 1985 | 0.911 | 1983 | 2 | 3.08 |
| Jamaica | 154.31 | -0.06 | 5.01 | -4.86 | 1984 | 1985 | 0.984 | 1984 | 1 | 4.95 |
| Japan | 875.90 | -0.41 | 0.22 | 0.78 | 1969 | 1995 | 0.778 | 1969 | 26 | -0.19 |
| Jordan | -1416.25 | 0.74 | -0.98 | 0.27 | 1977 | 1992 | 0.705 | 1977 | 15 | -0.24 |
| Kazakhstan** | - | - | - | - | - | - | - | - | - | - |
| Kenya | -628.79 | 0.34 | 5.66 | -5.90 | 1988 | 1993 | 0.997 | 1988 | 5 | 6.00 |
| Kiribati* | - | - | - | - | - | - | - | - | - | - |
| Kuwait | -1805.62 | 0.92 | -0.79 | -0.13 | 1989 | 2005 | 0.967 | 1989 | 16 | 0.13 |
| Kyrgyzstan | -301.69 | 0.18 | 1.53 | -1.67 | 1997 | 2003 | 0.970 | 1997 | 6 | 1.70 |
| Laos | 26.00 | 0.00 | 0.15 | -0.15 | 1974 | 2001 | 0.976 | 1974 | 27 | 0.15 |
| Latvia | 30.00 | 0.00 | 2.40 | -2.35 | 1995 | 2000 | 0.981 | 1995 | 5 | 2.40 |
| Lebanon | 103.07 | -0.02 | 0.46 | -0.41 | 1976 | 1990 | 0.939 | 1976 | 14 | 0.44 |
| Lesotho* | - | - | - | - | - | - | - | - | - | - |
| Liberia | 20.00 | 0.00 | 0.31 | -0.31 | 1978 | 1987 | 0.976 | 1978 | 9 | 0.31 |
| Libya | 330.79 | -0.15 | 9.15 | -9.00 | 1984 | 1985 | 0.965 | 1984 | 1 | 9.00 |
| Lithuania | -1233.34 | 0.63 | 3.85 | -4.36 | 2002 | 2004 | 0.978 | 2002 | 2 | 4.49 |
| Luxembourg | -3977.00 | 2.00 | -1.20 | -0.70 | 2001 | 2007 | 0.980 | 2001 | 6 | 0.80 |
| Madagascar | 57.00 | 0.00 | 0.40 | -0.40 | 1966 | 1993 | 0.971 | 1966 | 27 | 0.40 |
| Malawi | -292.43 | 0.16 | 0.89 | -0.94 | 1982 | 1992 | 0.994 | 1982 | 10 | 1.05 |
| Malaysia | 171.13 | -0.07 | 0.70 | -0.56 | 1983 | 1991 | 0.986 | 1983 | 8 | 0.63 |
| Maldives | -200.73 | 0.11 | -0.13 | 0.23 | 1974 | 1996 | 0.817 | 1974 | 22 | -0.02 |
| Mali** | - | - | - | - | - | - | - | - | - | - |
| Malta | -314.23 | 0.17 | 16.83 | -17.00 | 1997 | 1998 | 0.984 | 1997 | 1 | 17.00 |
| Marshall Is.* | - | - | - | - | - | - | - | - | - | - |
| Martinique | 143.95 | -0.06 | 0.70 | -0.65 | 1981 | 1996 | 0.964 | 1981 | 15 | 0.64 |
| Mauritania | 17.00 | 0.00 | 0.29 | -0.29 | 1989 | 1995 | 0.888 | 1989 | 6 | 0.29 |
| Mauritius | -79.43 | 0.06 | 0.64 | -0.67 | 1983 | 1988 | 0.975 | 1983 | 5 | 0.70 |
| Mexico | -98.38 | 0.09 | 1.86 | -1.92 | 1976 | 1991 | 0.982 | 1976 | 15 | 1.95 |
| Micronesia** | - | - | - | - | - | - | - | - | - | - |
| Mongolia | -23.05 | 0.02 | 0.62 | -0.56 | 1987 | 1998 | 0.983 | 1987 | 11 | 0.64 |
| Montenegro** | - | - | - | - | - | - | - | - | - | - |
| Montserrat** | - | - | - | - | - | - | - | - | - | - |
| Morocco | -141.36 | 0.10 | 19.90 | -19.96 | 1984 | 1985 | 0.998 | 1984 | 1 | 20.00 |
| Mozambique** | - | - | - | - | - | - | - | - | - | - |
| Myanmar | 37.00 | 0.00 | 0.31 | -0.31 | 1982 | 2001 | 0.989 | 1982 | 19 | 0.31 |
| N. Korea** | - | - | - | - | - | - | - | - | - | - |
| Namibia | -147.04 | 0.08 | 2.06 | -2.23 | 1995 | 2001 | 0.984 | 1995 | 6 | 2.14 |
| Nauru** | - | - | - | - | - | - | - | - | - | - |
| Nepal | -396.23 | 0.21 | 16.79 | -16.53 | 1989 | 1990 | 0.990 | 1989 | 1 | 17.00 |
| Netherlands | 138.08 | -0.05 | 1.91 | -1.77 | 1982 | 1990 | 0.990 | 1982 | 8 | 1.86 |
| New Caledonia** | - | - | - | - | - | - | - | - | - | - |
| New Zealand | -790.35 | 0.43 | -0.62 | 0.27 | 1986 | 1998 | 0.937 | 1986 | 12 | -0.19 |
| Nicaragua | -303.95 | 0.17 | 4.83 | -5.00 | 1990 | 1991 | 0.993 | 1990 | 1 | 5.00 |
| Niger | -230.68 | 0.13 | 7.37 | -7.31 | 1989 | 1991 | 0.995 | 1989 | 2 | 7.50 |
| Nigeria | 45.00 | 0.00 | 0.29 | -0.29 | 1979 | 1986 | 0.989 | 1979 | 7 | 0.29 |
| Niue** | - | - | - | - | - | - | - | - | - | - |
| Norway | -28.44 | 0.02 | 0.37 | -0.39 | 1976 | 2004 | 0.946 | 1976 | 28 | 0.39 |
| Oman | -185.89 | 0.10 | 0.42 | -0.49 | 1974 | 2007 | 0.971 | 1974 | 33 | 0.52 |
| Pakistan | -275.31 | 0.17 | 16.83 | -16.92 | 1979 | 1980 | 0.998 | 1979 | 1 | 17.00 |
| Panama | -55.97 | 0.04 | 1.79 | -1.82 | 1982 | 1988 | 0.983 | 1982 | 6 | 1.83 |
| Papua New Guinea | -234.60 | 0.13 | -0.08 | -0.06 | 1985 | 2008 | 0.937 | 1985 | 23 | 0.06 |
| Paraguay** | - | - | - | - | - | - | - | - | - | - |
| Peru | -1349.80 | 0.73 | -0.89 | 0.15 | 1976 | 1986 | 0.967 | 1976 | 10 | -0.16 |
| Philippines | -268.43 | 0.16 | 7.84 | -8.04 | 1989 | 1991 | 0.993 | 1989 | 2 | 8.00 |
| Poland | -36.43 | 0.03 | 2.21 | -1.83 | 1983 | 1992 | 0.982 | 1983 | 9 | 2.24 |
| Portugal | 115.30 | -0.03 | 3.03 | -3.03 | 1976 | 1978 | 0.878 | 1976 | 2 | 3.00 |
| Puerto Rico | 21.89 | 0.00 | 0.31 | 0.24 | 1977 | 1995 | 0.931 | 1977 | 18 | 0.31 |
| Qatar** | - | - | - | - | - | - | - | - | - | - |
| S. Korea | -371.45 | 0.22 | -1.27 | 1.13 | 1990 | 1993 | 0.645 | 1990 | 3 | -1.06 |
| Romania | 75.17 | -0.02 | 1.80 | -1.48 | 1981 | 1993 | 0.985 | 1981 | 12 | 1.78 |
| Russia | 61.00 | 0.00 | 0.50 | -0.83 | 1996 | 2009 | 0.955 | 1996 | 13 | 0.50 |
| Rwanda | -480.20 | 0.26 | -0.25 | 0.75 | 1971 | 1991 | 0.907 | 1971 | 20 | 0.00 |
| St. Kitts and Nevis | -77.55 | 0.04 | 1.21 | -1.26 | 1987 | 1994 | 0.985 | 1987 | 7 | 1.25 |
| Saint Lucia | 293.02 | -0.14 | 1.45 | -0.90 | 1978 | 1985 | 0.918 | 1978 | 7 | 1.31 |
| St. Vin. and Gren.** | - | - | - | - | - | - | - | - | - | - |
| Samoa | 21.00 | 0.00 | 0.00 | 0.00 | 2006 | 2015 | 0.500 | 2006 | 9 | 0.00 |
| Saudi Arabia | -304.80 | 0.17 | -0.54 | 0.43 | 1980 | 1986 | 0.735 | 1980 | 6 | -0.37 |
| Senegal | -173.09 | 0.10 | 0.42 | -0.52 | 1982 | 2003 | 0.981 | 1982 | 21 | 0.52 |
| Serbia | -1957.00 | 1.00 | -1.00 | 1.50 | 2007 | 2013 | 0.874 | 2007 | 6 | 0.00 |
| Seychelles | -168.52 | 0.09 | 0.16 | -0.25 | 1990 | 1996 | 0.978 | 1990 | 6 | 0.25 |
| Sierra Leone | -159.70 | 0.09 | 4.91 | -5.00 | 1984 | 1985 | 0.995 | 1984 | 1 | 5.00 |
| Singapore | 194.44 | -0.09 | -1.91 | 2.23 | 1995 | 1997 | 0.853 | 1995 | 2 | -2.00 |
| Slovakia | -496.68 | 0.28 | 1.21 | -1.50 | 2003 | 2005 | 0.946 | 2003 | 2 | 1.49 |
| Slovenia | 787.60 | -0.37 | 5.37 | -5.00 | 2002 | 2003 | 0.929 | 2002 | 1 | 5.00 |
| Solomon Is. | 16.00 | 0.00 | 0.08 | 0.02 | 1982 | 1993 | 0.860 | 1982 | 11 | 0.08 |
| Somalia | -338.76 | 0.18 | -0.07 | -0.11 | 1972 | 2004 | 0.962 | 1972 | 32 | 0.11 |
| South Africa | 65.00 | 0.00 | 0.27 | -0.27 | 1978 | 1992 | 0.982 | 1978 | 14 | 0.27 |
| Spain | -184.49 | 0.14 | 0.81 | -0.89 | 1980 | 1995 | 0.990 | 1980 | 15 | 0.95 |
| Sri Lanka | 43.00 | 0.00 | 0.21 | -0.20 | 1977 | 1991 | 0.981 | 1977 | 14 | 0.21 |
| Sudan | 153.21 | -0.06 | 0.58 | -0.52 | 1982 | 2006 | 0.981 | 1982 | 24 | 0.52 |
| Suriname | -100.15 | 0.06 | 0.54 | -0.46 | 1984 | 1988 | 0.979 | 1984 | 4 | 0.60 |
| Sweden | 328.32 | -0.15 | 1.18 | -1.02 | 1981 | 1989 | 0.952 | 1981 | 8 | 1.02 |
| Switzerland | -231.95 | 0.13 | 0.86 | -0.99 | 1979 | 2009 | 0.971 | 1979 | 30 | 0.99 |
| Syria | -1087.94 | 0.58 | -0.43 | -0.15 | 1968 | 2010 | 0.895 | 1968 | 42 | 0.15 |
| Taiwan | -440.43 | 0.26 | -0.42 | 0.17 | 1977 | 2004 | 0.842 | 1977 | 27 | -0.17 |
| Tajikistan | 1037.39 | -0.50 | 1.79 | -1.34 | 1994 | 1998 | 0.857 | 1994 | 4 | 1.29 |
| Thailand | -715.26 | 0.39 | -0.21 | -0.19 | 1991 | 2005 | 0.986 | 1991 | 14 | 0.19 |
| East Timor | 226.25 | -0.11 | 2.61 | -2.50 | 1988 | 1993 | 0.990 | 1988 | 5 | 2.50 |
| Togo | -62.84 | 0.04 | 0.66 | -0.59 | 1982 | 1993 | 0.990 | 1982 | 11 | 0.71 |
| Tokelau* | - | - | - | - | - | - | - | - | - | - |
| Trinidad and Tobago | 68.62 | -0.02 | 6.02 | -5.87 | 1984 | 1985 | 0.989 | 1984 | 1 | 6.00 |
| Tunisia | -111.62 | 0.08 | 2.66 | -2.63 | 1981 | 1990 | 0.994 | 1981 | 9 | 2.74 |
| Turkey | 85.17 | 0.00 | 0.15 | -0.25 | 1976 | 2008 | 0.949 | 1976 | 32 | 0.15 |
| Turkmenistan** | - | - | - | - | - | - | - | - | - | - |
| Tuvalu** | - | - | - | - | - | - | - | - | - | - |
| Uganda | 61.43 | -0.01 | 0.41 | -0.34 | 1985 | 1992 | 0.967 | 1985 | 7 | 0.39 |
| Ukraine | -3257.46 | 1.66 | -1.34 | -0.54 | 2002 | 2010 | 0.956 | 2002 | 8 | 0.32 |
| United Arab Emirates | -4785.69 | 2.44 | -1.76 | -0.58 | 1969 | 1979 | 0.966 | 1969 | 10 | 0.68 |
| United Kingdom | -197.76 | 0.12 | 0.52 | -0.63 | 1984 | 1992 | 0.984 | 1984 | 8 | 0.63 |
| Tanzania | -126.05 | 0.09 | 1.99 | -2.08 | 1992 | 1999 | 0.992 | 1992 | 7 | 2.08 |
| United States | 283.36 | -0.11 | 0.55 | -0.26 | 1976 | 1991 | 0.977 | 1976 | 15 | 0.44 |
| Uruguay** | - | - | - | - | - | - | - | - | - | - |
| Uzbekistan | 637.86 | -0.29 | 1.29 | -1.00 | 1998 | 2000 | 0.942 | 1998 | 2 | 1.00 |
| Vanuatu* | - | - | - | - | - | - | - | - | - | - |
| Venezuela | -59.19 | 0.05 | 2.45 | -2.31 | 1983 | 1986 | 0.981 | 1983 | 3 | 2.50 |
| Vietnam** | - | - | - | - | - | - | - | - | - | - |
| Wallis and Futuna** | - | - | - | - | - | - | - | - | - | - |
| W. Sahara* | - | - | - | - | - | - | - | - | - | - |
| Yemen | -184.34 | 0.10 | 22.90 | -22.84 | 1978 | 1979 | 0.990 | 1978 | 1 | 23.00 |
| Zambia | -874.60 | 0.45 | -0.22 | -0.24 | 1974 | 1993 | 0.985 | 1974 | 19 | 0.24 |
| Zimbabwe | -1355.27 | 0.70 | 14.30 | -15.01 | 1989 | 1990 | 0.992 | 1989 | 1 | 15.00 |

**Table S2.** Final piecewise modeling results for the 201 countries included in our analysis of temporal changes in crop evenness. Presented here are the same six parameters fitted for each country-specific piecewise model (see Equation 4), piecewise model r2 values, along with three indicators were calculated for each country (based on Equation 4, visualized in Figure 1). Piecewise models converged and were fitted to 183 countries. The 18 countries where model parameters, diagnostics, and indicators are denoted as “-”, represent the countries where piecewise models did not converge. In these instances, models for 7 countries did not converge because these countries reported no change in crop commodity group richness through time (denoted by “*” beside these country names). In the remaining 11 countries (denoted by “**” beside these country names) crop commodity group richness increased through time, between the first and last year of data. However, in these 11 countries increases in crop commodity group richness were primarily reflected by a single step increase.

|  | **Piecewise model parameters** | | | | | |  | **Indicators** | | |
| --- | --- | --- | --- | --- | --- | --- | --- | --- | --- | --- |
| **Country** | ***a*** | ***b*** | ***c*** | ***d*** | **ψ1** | **ψ2** | **Model *r*^2^** | **Indicator 1** | **Indicator 2** | **Indicator 3** |
| Afghanistan | -1.16 | 0.00 | 0.00 | 0.02 | 1985 | 2013 | 0.777 | 1985 | 28 | -0.003 |
| Albania | -4.46 | 0.00 | -0.02 | 0.03 | 1983 | 1994 | 0.941 | 1983 | 11 | -0.020 |
| Algeria | 7.97 | 0.00 | 0.01 | -0.01 | 1967 | 1988 | 0.658 | 1967 | 21 | 0.006 |
| American Samoa | -2.85 | 0.00 | 0.01 | -0.02 | 1998 | 2001 | 0.780 | 1998 | 3 | 0.016 |
| Angola | -0.99 | 0.00 | -0.01 | 0.01 | 1979 | 1988 | 0.792 | 1979 | 9 | -0.010 |
| Antigua and Barb.** | - | - | - | - | - | - | - | - | - | - |
| Argentina | -2.34 | 0.00 | -0.01 | 0.01 | 1979 | 2009 | 0.953 | 1979 | 30 | -0.007 |
| Armenia | 17.29 | -0.01 | 0.02 | -0.01 | 2003 | 2007 | 0.791 | 2003 | 4 | 0.015 |
| Australia | 6.33 | 0.00 | 0.01 | -0.01 | 1967 | 1995 | 0.845 | 1967 | 28 | 0.005 |
| Austria | 0.09 | 0.00 | 0.01 | -0.02 | 1986 | 1989 | 0.783 | 1986 | 3 | 0.015 |
| Azerbaijan | -1.71 | 0.00 | -0.01 | 0.02 | 2007 | 2012 | 0.301 | 2007 | 5 | -0.008 |
| Bahamas | -28.09 | 0.01 | -0.02 | 0.00 | 1968 | 1999 | 0.897 | 1968 | 31 | -0.005 |
| Bahrain | 10.31 | -0.01 | 0.03 | -0.04 | 1978 | 1994 | 0.902 | 1978 | 16 | 0.025 |
| Bangladesh | -5.10 | 0.00 | 0.00 | 0.01 | 1985 | 2010 | 0.895 | 1985 | 25 | 0.000 |
| Barbados | -12.84 | 0.01 | -0.01 | 0.03 | 1995 | 2010 | 0.924 | 1995 | 15 | -0.002 |
| Belarus | -26.48 | 0.01 | -0.01 | 0.00 | 2000 | 2008 | 0.957 | 2000 | 8 | 0.001 |
| Belgium | 11.26 | -0.01 | 0.00 | 0.00 | 1970 | 1979 | 0.733 | 1970 | 9 | -0.001 |
| Belize | -109.20 | 0.06 | -0.06 | 0.01 | 1962 | 1992 | 0.840 | 1962 | 30 | -0.005 |
| Benin | 9.43 | 0.00 | 0.02 | -0.01 | 1964 | 1969 | 0.430 | 1964 | 5 | 0.011 |
| Bermuda | -5.52 | 0.00 | -0.04 | 0.04 | 1982 | 1988 | 0.940 | 1982 | 6 | -0.036 |
| Bhutan | 4.82 | 0.00 | 0.00 | 0.00 | 1982 | 2006 | 0.620 | 1982 | 24 | 0.002 |
| Bolivia | -7.30 | 0.00 | 0.00 | 0.00 | 1972 | 1990 | 0.899 | 1972 | 18 | 0.000 |
| Bosnia and Herz. | 3.32 | 0.00 | -0.02 | 0.02 | 1995 | 1997 | 0.841 | 1995 | 2 | -0.022 |
| Botswana | -529.13 | 0.27 | -0.27 | 0.01 | 1962 | 1989 | 0.323 | 1962 | 27 | -0.002 |
| Brazil | -0.37 | 0.00 | 0.00 | -0.01 | 1973 | 2009 | 0.983 | 1973 | 36 | -0.002 |
| Brunei | -25.20 | 0.01 | -0.03 | 0.03 | 1979 | 1997 | 0.862 | 1979 | 18 | -0.015 |
| Bulgaria | 1.24 | 0.00 | -0.01 | 0.01 | 1985 | 2013 | 0.945 | 1985 | 28 | -0.006 |
| Burkina Faso | -0.56 | 0.00 | 0.00 | 0.01 | 1968 | 1993 | 0.876 | 1968 | 25 | -0.003 |
| Burundi | 6.01 | 0.00 | 0.00 | -0.01 | 1971 | 2009 | 0.725 | 1971 | 38 | 0.002 |
| Cambodia | -16.86 | 0.01 | -0.01 | 0.01 | 1974 | 1990 | 0.555 | 1974 | 16 | -0.005 |
| Cameroon | -5.02 | 0.00 | -0.01 | 0.00 | 1974 | 1986 | 0.659 | 1974 | 12 | -0.004 |
| Ca-da | 2.66 | 0.00 | 0.01 | -0.01 | 1992 | 2001 | 0.795 | 1992 | 9 | 0.009 |
| Cape Verde | -23.32 | 0.01 | -0.04 | 0.03 | 1977 | 1990 | 0.747 | 1977 | 13 | -0.026 |
| Central African Rep. | 4.59 | 0.00 | 0.01 | 0.00 | 1973 | 1994 | 0.804 | 1973 | 21 | 0.003 |
| Chad | -9.56 | 0.01 | -0.01 | 0.01 | 1982 | 2009 | 0.552 | 1982 | 27 | -0.002 |
| Chile | -7.30 | 0.00 | -0.01 | 0.01 | 1995 | 2002 | 0.862 | 1995 | 7 | -0.004 |
| Chi- | 6.18 | 0.00 | 0.00 | 0.00 | 1977 | 2007 | 0.804 | 1977 | 30 | 0.002 |
| Colombia | -8.15 | 0.00 | -0.01 | 0.00 | 1974 | 2011 | 0.772 | 1974 | 37 | -0.001 |
| Comoros | -4.90 | 0.00 | -0.01 | 0.01 | 1982 | 1992 | 0.975 | 1982 | 10 | -0.011 |
| Congo (Brazzaville) | -11.24 | 0.01 | -0.01 | 0.00 | 1972 | 1999 | 0.679 | 1972 | 27 | -0.001 |
| Cook Is. | -39.11 | 0.02 | -0.02 | -0.01 | 1978 | 2012 | 0.842 | 1978 | 34 | 0.001 |
| Costa Rica | -4.98 | 0.00 | -0.02 | 0.01 | 1980 | 1985 | 0.877 | 1980 | 5 | -0.013 |
| Ivory Coast** | - | - | - | - | - | - | - | - | - | - |
| Croatia | -6.41 | 0.00 | 0.00 | 0.01 | 1999 | 2013 | 0.809 | 1999 | 14 | 0.000 |
| Cuba | 1.13 | 0.00 | 0.01 | -0.02 | 1987 | 2008 | 0.957 | 1987 | 21 | 0.014 |
| Cyprus | -1.82 | 0.00 | 0.00 | 0.01 | 1977 | 2002 | 0.741 | 1977 | 25 | -0.003 |
| Czech Rep. | 4.83 | 0.00 | 0.01 | -0.01 | 2001 | 2003 | 0.791 | 2001 | 2 | 0.009 |
| Denmark | 18.53 | -0.01 | 0.05 | -0.04 | 1979 | 1985 | 0.923 | 1979 | 6 | 0.038 |
| Djibouti* | - | - | - | - | - | - | - | - | - | - |
| Dominica | 55.22 | -0.03 | 0.05 | -0.02 | 1966 | 1977 | 0.937 | 1966 | 11 | 0.018 |
| Dominican Rep. | 0.64 | 0.00 | 0.01 | -0.01 | 2010 | 2012 | 0.398 | 2010 | 2 | 0.012 |
| Ecuador | 0.91 | 0.00 | -0.01 | 0.01 | 1976 | 1986 | 0.854 | 1976 | 10 | -0.006 |
| Egypt | -4.45 | 0.00 | 0.00 | 0.00 | 1985 | 2007 | 0.911 | 1985 | 22 | 0.001 |
| El Salvador | -1.40 | 0.00 | 0.00 | 0.00 | 1977 | 1995 | 0.930 | 1977 | 18 | -0.002 |
| Eq. Guinea | -10.47 | 0.01 | 0.01 | -0.01 | 1987 | 2003 | 0.969 | 1987 | 16 | 0.013 |
| Eritrea | 9.72 | 0.00 | -0.04 | 0.05 | 2004.882 | 2006.427 | 0.861 | 2004.882 | 2 | 0.079 |
| Estonia | -24.71 | 0.01 | -0.04 | 0.03 | 2003 | 2006 | 0.829 | 2003 | 3 | -0.025 |
| Ethiopia | -1.30 | 0.00 | -0.03 | 0.03 | 1992 | 1996 | 0.921 | 1992 | 4 | -0.027 |
| Faroe Is.* | - | - | - | - | - | - | - | - | - | - |
| Fiji | 21.47 | -0.01 | 0.04 | -0.03 | 1981 | 1985 | 0.788 | 1981 | 4 | 0.029 |
| Finland | 1.98 | 0.00 | 0.00 | 0.01 | 1989 | 1997 | 0.643 | 1989 | 8 | -0.005 |
| France | 3.68 | 0.00 | 0.01 | 0.00 | 1981 | 1986 | 0.839 | 1981 | 5 | 0.003 |
| French Guiana | -17.54 | 0.01 | -0.03 | 0.03 | 1975 | 1995 | 0.843 | 1975 | 20 | -0.017 |
| Fr. Polynesia | 16.48 | -0.01 | 0.01 | 0.00 | 1966 | 2005 | 0.625 | 1966 | 39 | 0.002 |
| Gabon | 5.83 | 0.00 | 0.02 | -0.02 | 1980 | 1982 | 0.766 | 1980 | 2 | 0.014 |
| Gambia | 0.56 | 0.00 | -0.02 | 0.02 | 1998 | 2000 | 0.300 | 1998 | 2 | -0.020 |
| Georgia | 20.59 | -0.01 | -0.01 | 0.02 | 1997 | 1998 | 0.766 | 1997 | 1 | -0.020 |
| Germany | 3.08 | 0.00 | 0.00 | 0.00 | 1978 | 1997 | 0.819 | 1978 | 19 | 0.001 |
| Ghana | -28.76 | 0.01 | -0.02 | 0.00 | 1974 | 2011 | 0.898 | 1974 | 37 | -0.001 |
| Greece | 1.40 | 0.00 | 0.00 | 0.00 | 1989 | 2005 | 0.635 | 1989 | 16 | -0.002 |
| Grenada | 11.07 | -0.01 | 0.01 | -0.03 | 1976 | 2011 | 0.803 | 1976 | 35 | 0.006 |
| Guadeloupe | -0.49 | 0.00 | 0.03 | -0.02 | 1982 | 1985 | 0.891 | 1982 | 3 | 0.025 |
| Guam | 23.38 | -0.01 | -0.49 | 0.50 | 1984 | 1985 | 0.990 | 1984 | 1 | -0.502 |
| Guatemala** | - | - | - | - | - | - | - | - | - | - |
| Guinea | 3.27 | 0.00 | 0.00 | -0.01 | 1972 | 2006 | 0.963 | 1972 | 34 | -0.002 |
| Guinea Bissau | -18.31 | 0.01 | -0.01 | 0.00 | 1968 | 1990 | 0.908 | 1968 | 22 | -0.002 |
| Guyana | -4.16 | 0.00 | 0.08 | -0.09 | 1984 | 1985 | 0.773 | 1984 | 1 | 0.084 |
| Haiti | -6.97 | 0.00 | -0.01 | 0.01 | 1983 | 1995 | 0.782 | 1983 | 12 | -0.006 |
| Honduras | -5.02 | 0.00 | -0.01 | 0.01 | 1983 | 1992 | 0.609 | 1983 | 9 | -0.008 |
| Hungary | 11.17 | -0.01 | 0.01 | 0.00 | 1974 | 1989 | 0.903 | 1974 | 15 | 0.002 |
| Iceland | -0.29 | 0.00 | 0.01 | -0.01 | 1991 | 1999 | 0.719 | 1991 | 8 | 0.011 |
| India | 0.00 | 0.00 | 0.00 | 0.00 | 1980 | 2012 | 0.968 | 1980 | 32 | 0.001 |
| Indonesia | 2.46 | 0.00 | 0.00 | -0.01 | 1966 | 2008 | 0.874 | 1966 | 42 | 0.001 |
| Iran | -2.23 | 0.00 | 0.01 | -0.01 | 1988 | 1999 | 0.923 | 1988 | 11 | 0.008 |
| Iraq | -11.48 | 0.01 | 0.00 | -0.01 | 1966 | 2009 | 0.348 | 1966 | 43 | 0.002 |
| Ireland | 3.51 | -0.01 | 0.02 | -0.01 | 1982 | 1994 | 0.929 | 1982 | 12 | 0.009 |
| Israel | 1.59 | 0.00 | 0.01 | -0.01 | 1981 | 2000 | 0.923 | 1981 | 19 | 0.005 |
| Italy | 1.51 | 0.00 | 0.00 | 0.00 | 1982 | 1993 | 0.835 | 1982 | 11 | 0.004 |
| Jamaica | -10.34 | 0.01 | -0.01 | 0.01 | 1989 | 2007 | 0.918 | 1989 | 18 | -0.003 |
| Japan | 9.00 | 0.00 | 0.02 | -0.02 | 1977 | 1981 | 0.717 | 1977 | 4 | 0.014 |
| Jordan | 31.24 | -0.02 | 0.03 | -0.01 | 1971 | 1990 | 0.871 | 1971 | 19 | 0.010 |
| Kazakhstan | 35.97 | -0.02 | 0.02 | 0.02 | 1998 | 2008 | 0.969 | 1998 | 10 | -0.002 |
| Kenya | 12.65 | -0.01 | 0.01 | 0.00 | 1968 | 1982 | 0.602 | 1968 | 14 | 0.004 |
| Kiribati | -10.97 | 0.01 | -0.01 | 0.01 | 1976 | 1986 | 0.904 | 1976 | 10 | -0.005 |
| Kuwait | 24.54 | -0.01 | 0.03 | -0.02 | 1978 | 1988 | 0.777 | 1978 | 10 | 0.017 |
| Kyrgyzstan | -2.66 | 0.00 | 0.01 | -0.01 | 1992 | 2001 | 0.795 | 1992 | 9 | 0.009 |
| Laos | 15.49 | -0.01 | 0.02 | 0.00 | 1965 | 1992 | 0.978 | 1965 | 27 | 0.008 |
| Latvia | -6.08 | 0.00 | -0.02 | 0.02 | 2005 | 2011 | 0.967 | 2005 | 6 | -0.020 |
| Lebanon | -5.91 | 0.00 | -0.02 | 0.02 | 1996 | 2001 | 0.886 | 1996 | 5 | -0.016 |
| Lesotho | -8.48 | 0.00 | -0.03 | 0.03 | 1977 | 1980 | 0.435 | 1977 | 3 | -0.027 |
| Liberia | -2.96 | 0.00 | 0.04 | -0.04 | 1990 | 1993 | 0.780 | 1990 | 3 | 0.038 |
| Libya | -16.21 | 0.01 | 0.01 | -0.02 | 1982 | 1993 | 0.959 | 1982 | 11 | 0.018 |
| Lithuania | -26.37 | 0.01 | -0.02 | 0.01 | 1999 | 2006 | 0.944 | 1999 | 7 | -0.011 |
| Luxembourg | 15.18 | -0.01 | -0.04 | 0.05 | 2006 | 2007 | 0.982 | 2006 | 1 | -0.045 |
| Macedonia | 1.18 | 0.00 | 0.01 | -0.01 | 2005 | 2011 | 0.821 | 2005 | 6 | 0.008 |
| Madagascar | 0.93 | 0.00 | 0.00 | 0.01 | 1977 | 2010 | 0.943 | 1977 | 33 | 0.001 |
| Malawi | -3.04 | 0.00 | -0.01 | 0.01 | 1980 | 1990 | 0.701 | 1980 | 10 | -0.004 |
| Malaysia | -9.52 | 0.01 | -0.01 | 0.01 | 1987 | 2010 | 0.992 | 1987 | 23 | -0.008 |
| Maldives** | - | - | - | - | - | - | - | - | - | - |
| Mali | -6.19 | 0.00 | -0.01 | 0.00 | 1971 | 1989 | 0.747 | 1971 | 18 | -0.004 |
| Malta | 1.18 | 0.00 | -0.09 | 0.08 | 1997 | 1998 | 0.982 | 1997 | 1 | -0.085 |
| Marshall Is.* | - | - | - | - | - | - | - | - | - | - |
| Martinique | -15.31 | 0.01 | -0.02 | 0.02 | 1985 | 1995 | 0.697 | 1985 | 10 | -0.017 |
| Mauritania | -26.05 | 0.01 | -0.08 | 0.06 | 1980 | 1981 | 0.781 | 1980 | 1 | -0.062 |
| Mauritius | 13.82 | -0.01 | 0.01 | 0.01 | 1964 | 2006 | 0.859 | 1964 | 42 | 0.000 |
| Mexico | -8.74 | 0.00 | -0.01 | 0.01 | 1979 | 1994 | 0.755 | 1979 | 15 | -0.004 |
| Micronesia | -0.91 | 0.00 | -0.01 | 0.02 | 2009 | 2015 | 0.787 | 2009 | 6 | -0.006 |
| Mongolia | -11.31 | 0.01 | -0.05 | 0.05 | 1987 | 1994 | 0.803 | 1987 | 7 | -0.041 |
| Montenegro* | - | - | - | - | - | - | - | - | - | - |
| Montserrat | -47.98 | 0.02 | -0.02 | 0.00 | 1969 | 1992 | 0.934 | 1969 | 23 | 0.006 |
| Morocco* | - | - | - | - | - | - | - | - | - | - |
| Mozambique | -2.07 | 0.00 | -0.01 | 0.01 | 1976 | 1988 | 0.843 | 1976 | 12 | -0.007 |
| Myanmar | -17.93 | 0.01 | -0.01 | 0.00 | 1963 | 1994 | 0.914 | 1963 | 31 | 0.002 |
| N. Korea | 3.16 | 0.00 | 0.00 | 0.00 | 1977 | 2003 | 0.966 | 1977 | 26 | 0.003 |
| Namibia | 7.92 | 0.00 | -0.04 | 0.04 | 1993 | 1997 | 0.920 | 1993 | 4 | -0.039 |
| Nauru | -4.12 | 0.00 | 0.01 | 0.00 | 1966 | 2005 | 0.966 | 1966 | 39 | 0.008 |
| Nepal | -4.84 | 0.00 | -0.01 | 0.01 | 1984 | 1999 | 0.867 | 1984 | 15 | -0.007 |
| Netherlands | 7.32 | 0.00 | 0.01 | 0.00 | 1996 | 2003 | 0.925 | 1996 | 7 | 0.003 |
| New Caledonia** | - | - | - | - | - | - | - | - | - | - |
| New Zealand | 11.80 | -0.01 | 0.01 | -0.01 | 1969 | 2000 | 0.900 | 1969 | 31 | 0.006 |
| Nicaragua | -4.73 | 0.00 | -0.01 | 0.01 | 1988 | 2003 | 0.789 | 1988 | 15 | -0.008 |
| Niger | -9.04 | 0.00 | -0.01 | 0.01 | 1967 | 1995 | 0.874 | 1967 | 28 | -0.004 |
| Nigeria | -7.26 | 0.00 | -0.02 | 0.02 | 1983 | 1986 | 0.843 | 1983 | 3 | -0.014 |
| Niue | -38.16 | 0.02 | -0.04 | 0.02 | 1980 | 1993 | 0.886 | 1980 | 13 | -0.016 |
| Norway** | - | - | - | - | - | - | - | - | - | - |
| Oman | -1.46 | 0.00 | -0.01 | 0.06 | 1968 | 2013 | 0.905 | 1968 | 45 | -0.005 |
| Pakistan | 7.10 | 0.00 | 0.00 | 0.00 | 1986 | 2007 | 0.897 | 1986 | 21 | 0.000 |
| Pa-ma | -8.19 | 0.00 | 0.00 | -0.01 | 1986 | 2014 | 0.900 | 1986 | 28 | 0.002 |
| Papua New Guinea | -12.27 | 0.01 | 0.00 | 0.00 | 1974 | 2004 | 0.984 | 1974 | 30 | 0.003 |
| Paraguay | -0.92 | 0.00 | -0.02 | 0.01 | 1974 | 1979 | 0.982 | 1974 | 5 | -0.016 |
| Peru | -2.92 | 0.00 | 0.00 | 0.00 | 1993 | 2005 | 0.925 | 1993 | 12 | -0.001 |
| Philippines | 2.77 | 0.00 | 0.00 | 0.00 | 1968 | 1990 | 0.363 | 1968 | 22 | 0.000 |
| Poland | -2.33 | 0.00 | -0.01 | 0.01 | 1989 | 1994 | 0.696 | 1989 | 5 | -0.011 |
| Portugal | -5.92 | 0.00 | 0.00 | 0.00 | 1973 | 1993 | 0.923 | 1973 | 20 | 0.001 |
| Puerto Rico | -2.98 | 0.00 | -0.01 | 0.02 | 1992 | 2003 | 0.772 | 1992 | 11 | -0.009 |
| Qatar** | - | - | - | - | - | - | - | - | - | - |
| S. Korea | -7.99 | 0.00 | 0.00 | 0.00 | 1969 | 2009 | 0.630 | 1969 | 40 | 0.000 |
| Romania | -2.35 | 0.00 | -0.03 | 0.02 | 1989 | 1993 | 0.961 | 1989 | 4 | -0.024 |
| Russia | 6.77 | 0.00 | 0.03 | -0.02 | 2009 | 2012 | 0.807 | 2009 | 3 | 0.022 |
| Rwanda | -0.06 | 0.00 | 0.00 | 0.00 | 1980 | 2000 | 0.572 | 1980 | 20 | -0.002 |
| St. Kitts and Nevis | -9.41 | 0.00 | -0.01 | 0.03 | 1989 | 1999 | 0.764 | 1989 | 10 | -0.006 |
| Saint Lucia | 97.76 | -0.05 | 0.08 | -0.02 | 1965 | 1971 | 0.860 | 1965 | 6 | 0.026 |
| St. Vin. and Gren. | -0.99 | 0.00 | 0.01 | -0.02 | 1992 | 2003 | 0.622 | 1992 | 11 | 0.009 |
| Samoa** | - | - | - | - | - | - | - | - | - | - |
| Sao Tome and Principe | 4.86 | 0.00 | 0.01 | -0.02 | 1978 | 2006 | 0.944 | 1978 | 28 | 0.010 |
| Saudi Arabia | 10.84 | -0.01 | 0.01 | -0.01 | 1988 | 2013 | 0.634 | 1988 | 25 | 0.009 |
| Senegal | 0.30 | 0.00 | 0.00 | 0.00 | 1981 | 2010 | 0.766 | 1981 | 29 | 0.003 |
| Serbia | -4.72 | 0.00 | -0.01 | 0.02 | 2009 | 2015 | 0.747 | 2009 | 6 | -0.009 |
| Seychelles | 9.07 | 0.00 | 0.01 | 0.01 | 1968 | 2002 | 0.821 | 1968 | 34 | 0.005 |
| Sierra Leone | 13.42 | -0.01 | 0.02 | -0.01 | 1977 | 1996 | 0.857 | 1977 | 19 | 0.010 |
| Singapore | 8.38 | 0.00 | -0.08 | 0.10 | 1991 | 1996 | 0.969 | 1991 | 5 | -0.089 |
| Slovakia | 1.92 | 0.00 | -0.01 | 0.01 | 2005 | 2013 | 0.886 | 2005 | 8 | -0.008 |
| Slovenia* | - | - | - | - | - | - | - | - | - | - |
| Solomon Is. | -14.66 | 0.01 | 0.00 | 0.00 | 1982 | 2002 | 0.943 | 1982 | 20 | 0.003 |
| Somalia | -15.96 | 0.01 | -0.01 | 0.01 | 1969 | 2010 | 0.842 | 1969 | 41 | 0.003 |
| South Africa | 0.71 | 0.00 | 0.00 | 0.00 | 1983 | 2002 | 0.861 | 1983 | 19 | 0.003 |
| S. Sudan | 0.71 | 0.00 | 0.00 | 0.00 | 1983 | 2002 | 0.861 | 1983 | 19 | 0.003 |
| Spain | -2.43 | 0.00 | 0.00 | 0.00 | 1975 | 2008 | 0.522 | 1975 | 33 | -0.001 |
| Sri Lanka | -3.25 | 0.00 | 0.00 | 0.01 | 1987 | 2012 | 0.607 | 1987 | 25 | -0.002 |
| Sudan | -30.14 | 0.02 | -0.02 | 0.01 | 1964 | 2005 | 0.651 | 1964 | 41 | -0.003 |
| Suri-me | 18.88 | -0.01 | 0.02 | -0.01 | 1982 | 1991 | 0.862 | 1982 | 9 | 0.008 |
| Sweden | 14.51 | -0.01 | 0.01 | 0.00 | 1966 | 1974 | 0.198 | 1966 | 8 | 0.003 |
| Switzerland | -8.66 | 0.00 | -0.01 | 0.00 | 1977 | 1992 | 0.893 | 1977 | 15 | -0.004 |
| Syria | -2.61 | 0.00 | -0.04 | 0.04 | 1987 | 1989 | 0.660 | 1987 | 2 | -0.040 |
| Taiwan | -5.74 | 0.00 | 0.00 | -0.01 | 1978 | 1994 | 0.974 | 1978 | 16 | 0.007 |
| Tajikistan | 22.39 | -0.01 | 0.01 | 0.01 | 1998 | 2001 | 0.921 | 1998 | 3 | 0.002 |
| Thailand | -15.70 | 0.01 | -0.01 | 0.00 | 1972 | 1992 | 0.924 | 1972 | 20 | 0.002 |
| East Timor | -9.08 | 0.01 | -0.02 | 0.01 | 1973 | 1993 | 0.966 | 1973 | 20 | -0.012 |
| Togo | -7.29 | 0.00 | -0.01 | 0.01 | 1981 | 1989 | 0.891 | 1981 | 8 | -0.010 |
| Tokelau | -10.80 | 0.01 | -0.02 | 0.01 | 1980 | 1983 | 0.446 | 1980 | 3 | -0.014 |
| Trinidad and Tobago | -2.03 | 0.00 | 0.01 | -0.02 | 2001 | 2011 | 0.912 | 2001 | 10 | 0.015 |
| Tunisia** | - | - | - | - | - | - | - | - | - | - |
| Turkey | 0.95 | 0.00 | 0.00 | 0.00 | 1970 | 2010 | 0.865 | 1970 | 40 | 0.001 |
| Turkmenistan** | - | - | - | - | - | - | - | - | - | - |
| Tuvalu | -5.52 | 0.00 | 0.02 | -0.02 | 1983 | 1987 | 0.925 | 1983 | 4 | 0.027 |
| Uganda | 1.14 | 0.00 | 0.00 | 0.00 | 1990 | 2010 | 0.752 | 1990 | 20 | 0.002 |
| Ukraine | 13.98 | -0.01 | 0.01 | 0.00 | 2002 | 2012 | 0.926 | 2002 | 10 | -0.001 |
| United Arab Emirates | -5.33 | 0.00 | -0.04 | 0.05 | 1986 | 2005 | 0.903 | 1986 | 19 | -0.033 |
| United Kingdom | 5.36 | 0.00 | 0.01 | -0.01 | 1981 | 1993 | 0.766 | 1981 | 12 | 0.006 |
| Tanzania | -3.20 | 0.00 | 0.00 | 0.00 | 1976 | 2008 | 0.568 | 1976 | 32 | -0.002 |
| United States | 6.06 | 0.00 | 0.00 | 0.00 | 1980 | 1994 | 0.961 | 1980 | 14 | -0.001 |
| Uruguay | -9.82 | 0.01 | -0.01 | -0.01 | 1984 | 2003 | 0.925 | 1984 | 19 | -0.004 |
| Uzbekistan | 19.72 | -0.01 | 0.01 | 0.01 | 1997 | 2004 | 0.905 | 1997 | 7 | -0.001 |
| Vanuatu | -10.72 | 0.01 | -0.01 | 0.01 | 1991 | 2008 | 0.955 | 1991 | 17 | -0.002 |
| Venezuela | -6.94 | 0.00 | 0.00 | 0.02 | 1980 | 2013 | 0.418 | 1980 | 33 | -0.001 |
| Viet-m | 0.37 | 0.00 | 0.00 | 0.00 | 1969 | 2007 | 0.964 | 1969 | 38 | 0.005 |
| Wallis and Futuna** | - | - | - | - | - | - | - | - | - | - |
| W. Sahara* | - | - | - | - | - | - | - | - | - | - |
| Yemen | -9.72 | 0.01 | 0.01 | -0.01 | 1988 | 1997 | 0.892 | 1988 | 9 | 0.011 |
| Zambia | 0.99 | 0.00 | 0.01 | -0.01 | 1973 | 2002 | 0.937 | 1973 | 29 | 0.008 |
| Zimbabwe | -10.85 | 0.01 | -0.01 | 0.01 | 1973 | 2005 | 0.633 | 1973 | 32 | -0.003 |

**Figure S1.** Visualization of the differences in crop group composition among countries from 1961, 1983, and 2017 based on non-metric multidimensional scaling (NMDS) analysis. Coloured symbols correspond to data for individual years, with only data for three years presented here for clarity, including the first year of data availability for the majority of countries (1961), the average year in which diversity begins to change across all countries (1983, see Figure 2A and B in the main text), and the final year of data availability (2017). Lines correspond to 95% confidence ellipses surrounding the data in a given year, and statistical results are presented in Table 2 in the main text.
